# Incidence rates of the most common canine tumors based on data from the Swiss Canine Cancer Registry (2008 to 2020)

**DOI:** 10.1371/journal.pone.0302231

**Published:** 2024-04-18

**Authors:** Elena Sophie Dhein, Ulla Heikkilä, Anna Oevermann, Sohvi Blatter, Daniela Meier, Sonja Hartnack, Franco Guscetti

**Affiliations:** 1 Institute of Veterinary Pathology, Vetsuisse Faculty, University of Zurich, Zurich, Switzerland; 2 Identitas AG, Bern, Switzerland; 3 Department of Clinical Research and Veterinary Public Health, Division of Neurological Sciences, Vetsuisse Faculty, University of Bern, Bern, Switzerland; 4 Institute of Animal Pathology, Vetsuisse Faculty, University of Bern, Bern, Switzerland; 5 Zyto/Histo Diagnostik Labor Freienstein, Freienstein, Switzerland; 6 Section of Epidemiology, Vetsuisse Faculty, University of Zurich, Zurich, Switzerland; Colorado State University, UNITED STATES

## Abstract

Monitoring neoplasms in standardized registries facilitates epidemiologic studies of risk factors for tumor development and predisposition. In an observational study, we determined incidence rates (IR) and malignant tumor incidence rate ratios (IRR) by age, sex, and breed in Swiss dogs using demographic data from the official Swiss dog registration database Amicus. The dataset analyzed included 54’986 tumors diagnosed by histology and cytology in four Swiss veterinary pathology laboratories between 2008 and 2020. Diagnoses were coded according to the Vet-ICD-O-canine-1 system. Most tumors occurred in the skin (n = 19’045; 34.64%), soft tissues (n = 11’092; 20.17%), and mammary glands (n = 7’974; 14.50%). The IRs for all and for malignant tumors were 775/100’000 dog-years at risk (95%CI 764–777) and 338/100’000 dog-years at risk (95%CI 333–342), respectively. Females (850; 95%CI 834–853) had a higher overall tumor IR than males (679; 95%CI 666–684). The highest tumor IR was found at 11 years of age (1’857; 95%CI 1’780–1’867). Potential novel breed-specific predispositions were uncovered, with high IRs for several benign and malignant tumors in Polski Owczarek Nizinnys (overall IR: 3’303; 95%CI 2’502–3’864) and high IRs for malignant tumors in Russian Black Terriers (melanomas: 345; 95%CI 138–708), Field Spaniels (adenocarcinomas: 376; CI95% 138–817), Dogo Argentinos (mast cell tumors: 844; CI95% 591–1‘169), King Charles Spaniels and Manchester Terriers (lymphomas: 319; CI95% 137–627 and 302; CI95% 98–704, respectively), Landseers (osteosarcomas: 74; CI95% 15–216), Bouvier des Flandres (hemangiosarcomas: 127; CI95% 26–371), and Bearded Collies and Cane Corso Italianos (gliomas: 91; CI95% 45–162 and 34; CI95% 7–99, respectively). Nordic hunting dogs had the highest (8.08; CI95% 3.55–16.7) and Chihuahueno the lowest cancer IRRs (0.42; 95%CI 0.31–0.57) compared to mixed breeds. In conclusion, the calculated IRs and IRRs revealed previously unknown predisposing factors, including novel breed-specific susceptibilities. The results may have implications for cancer screening, diagnostic work-up, breeding management and oncologic and translational research.

## Introduction

Cancer is the leading cause of death in dogs, accounting for 27% [1] of all mortalities, and for more than 30% in dogs over one year of age [2]. The documentation of disease cases in cancer registries is essential for monitoring cancer burden [3] and provides the basis for epidemiological research. Furthermore, cancer registry data facilitates the identification of risk factors for tumor development.

In addition to age [4–8] and sex [4, 8–11], breed may also be an indicator of tumor susceptibility and predispositions to certain types of neoplasms have been described in specific dog breeds [4, 8, 12–15]. A better understanding of tumorigenesis can be achieved by investigating the underlying mechanisms of predisposition, which may ultimately contribute to the development of effective tumor prevention strategies.

Ideally, a cancer registry includes all tumors diagnosed within a population over a defined period of time. Accurate quantification of tumor incidence depends on a well-characterized reference population, requiring precise demographic data. Comparability between studies can be achieved through uniform inclusion criteria, collection of standardized data, and consistent data analysis [3].

Data from several epidemiological studies have provided insights on the occurrence of cancer in dogs [4, 5, 7–11, 15–28], some of which were derived from cancer registries [4, 5, 9–11, 22–24, 26]. The time periods evaluated in these studies ranged from 12 months [16] to 18 years [9]. The number of tumors recorded varied from 899 to 28’727 [4, 5, 7–11, 16–21, 24–28]. Two studies from the Swiss Canine Cancer Registry (SCCR) collected 63’214 tumors from 1955 to 2008 [22, 23] and one recent study from Germany comprised 70’966 neoplasms [15]. To classify tumors, several studies applied the World Health Organization (WHO) International Classification of Diseases for Oncology (ICD-O) guidelines [4, 8–11, 15, 18, 19, 22, 23, 27], a coding system developed for neoplasms in humans that has been continuously updated and therefore modified over time. Other surveys on tumors in dogs have not used an official system to categorize neoplasms [5, 16, 17, 20, 21, 24, 25, 28]. A novel international guideline for coding canine tumors was established by the Global Initiative for Veterinary Cancer Surveillance (GIVCS). Recently, the international tumor coding system Vet-ICD-O-canine-1 [29] was published and applied to 7’355 neoplasms in a Portuguese study [7]. From the surveys mentioned above, some have calculated tumor incidence rates (IR) [4, 9, 10, 16, 19, 23, 24], while others have examined incidence [11, 21], prevalence [20, 27], risk ratios (RR) [17], odds ratios (OR) [7, 15, 22, 25, 28], proportional morbidity ratios (PMR) [8], or standard morbidity ratios (SMR) [5]. Despite the fact that the studies differ in design, they have come to similar conclusions consistently revealing tumor predispositions in dogs. However, the calculation of IRs from cancer registries and demographic data has been described as the state-of-the-art method [30].

A previous study from Switzerland analyzed 11’740 cutaneous tumors recorded in the SCCR between 2008 and 2013 (hereafter referred to as “SCCR cutaneous study”). Tumors were coded according to the ICD-O-3 guidelines and IRs were calculated based on the Swiss dog registration database ANIS [4]. The aim of the present study was to investigate the epidemiology of canine tumors in Switzerland between 2008 and 2020 and to identify animal-related factors influencing tumorigenesis and biological tumor behavior. To this end, we examined 54’986 tumors diagnosed in four Swiss veterinary laboratories over a 13-year period and coded according to the new Vet-ICD-O-canine-1 coding system. Overall tumor IRs and IRs for benign and malignant tumors were calculated by age, sex, and breed using official Swiss dog registration data. Our analysis was extended by a negative binomial regression model providing incidence rate ratios (IRR) for malignant tumors. This is the largest study to date of breed-specific IRs for neoplasms in dogs, and it reveals previously unknown breed predispositions. Our findings expand the knowledge of tumor susceptibility in dogs, with potential implications for several areas of veterinary medicine, including cancer screening, diagnostic work-up, breeding management, and translational and cancer research in the context of One Health.

## Materials and methods

This study was designed as a historical open cohort study based on tumor data from the SCCR, including all tumors diagnosed between January 2008 and December 2020 [31] by four different veterinary pathology laboratories in Switzerland. Diagnoses were retrospectively evaluated and overall tumor IRs, IRs for benign and malignant tumors, and IRRs for malignant tumors were determined using the Swiss dog population as a reference.

### Data source

Tumor data were provided by four veterinary pathology laboratories in Switzerland: the Institute of Veterinary Pathology of the Vetsuisse Faculty Zurich; the Institute of Animal Pathology of the Vetsuisse Faculty Bern; the Division of Neurological Sciences, Department of Clinical Research and Veterinary Public Health of the Vetsuisse Faculty Bern; and the laboratory Zyto/Histo Diagnostik Freienstein, Switzerland.

Data evaluated included cases analyzed by histology (including biopsy and necropsy samples) and cytology by board or nationally certified veterinary pathologists. For cases with more than one type of diagnostic analysis performed, after manual review, the most accurate morphological diagnosis and respective metadata were considered, while date and age at first pathological diagnosis were retained. The remaining results were removed from the dataset.

Data on the general dog population were provided by Identitas AG, which has been operating the national Swiss dog database Amicus since 2016. The mandatory registration of dogs in Switzerland is based on the legal requirements of the Law on Epizootic Diseases (Tierseuchengesetz, TSG) 916.40, article 30 [32], which has been in force since July 2008. Before 2016, dogs in Switzerland were registered in the ANIS (Animal Identity Service) database. For this study, Identitas AG provided a combined dataset of ANIS and Amicus data with dogs registered in Switzerland, including the Principality of Liechtenstein, between January 1991 and December 2020, and alive between January 2008 and December 2020. The dataset is available upon request from Identitas AG (info@identitas.ch). It was not possible to directly link the individual dogs in this dataset to those in the tumor dataset.

### Data preparation

Data was cleaned and managed using Microsoft Excel version 16.69 (Microsoft Corporation 2022) and R Statistical Software version 4.1.2 [33].

#### Case inclusion

To identify dogs with neoplasms for the present study, the diagnosis field in the analytic dataset was searched for tumor diagnoses by filtering for keywords (S1 Table). Diagnosis texts containing a keyword were manually reviewed to determine case inclusion or exclusion. Inclusion criteria required evidence in the pathology report that the dog lived in Switzerland and was diagnosed with at least one tumor according to the Vet-ICD-O-canine-1 coding system [29] at any time between January 1^st^, 2008, and December 31^st^, 2020. Cases that did not meet these criteria as well as records reporting a tentative diagnosis, a diagnosis of exclusion, or a non-neoplastic differential diagnosis were removed and excluded from the analysis. As an exception, peripheral odontogenic fibroma was considered as a tumor [14] and was therefore included in the group of *odontogenic tumors [927–934]*, using the ICD-O-3.2 [34] term and code *peripheral odontogenic fibroma [9322/0]*. The diagnoses were named, coded, and categorized according to the tumor morphology (tumor type) and topography (anatomical body location of a tumor) lists of the Vet-ICD-O-canine-1 coding system [35]. All tumor cases were included, even if one or more parameters (e.g., age, sex, breed, or topography) were missing. For the purpose of this work, morphological tumor groups and categories variably defined by the Vet-ICD-O-canine-1 are referred to here as tumor “categories”. Groups created out of these categories based on their postulated biological behavior are referred to as tumor “groups”.

#### Data processing

The tumor behavior codes *benign [/0]* to *malignant [/3]* were adopted from the corresponding tumor code indicated in the Vet-ICD-O-canine-1. If this code did not match the behavior described in the diagnosis text, it was adjusted according to the definitions of Vet-ICD-O-canine-1. For further analysis, tumors of *uncertain behavior [/1]* and *in situ neoplasms [/2]* were added either to the *[/0]* tumors to form a group of benign tumors, or to the *[/3]* tumors to form a group of malignant neoplasms. This reassignment was made where deemed appropriate and based on the currently available literature, as detailed in S2 Table. In both case and reference populations, dogs with more than one breed listed, mixed breeds, and crosses such as Labradoodles, Goldendoodles, and Puggles were classified as “mixed breed”. Dogs with no breed information were classified as “unknown breed”. Commonly accepted breed varieties (e.g., Schnauzer—Giant) were retained, while the remaining individuals were categorized as “not otherwise specified” (NOS), e.g., Schnauzer NOS. These entities were treated individually as “precise breeds” and together as “grouped breeds” for calculations.

For some tumors, explicit topography information was missing, or the tissue of origin was imprecisely described as "skin/subcutis". These tumors were topographically assigned to their tissue of origin, where possible, after a plausibility check for each case, considering tumor morphology and topography codes indicated in the Vet-ICD-O-canine-1. Cases that could not be assigned to a specific tissue with sufficient certainty were topographically classified as *other and ill-defined sites [C76]*. The morphological group of *nerve sheath tumors [954–957]*, when found in the skin, soft tissue, or bone, was assigned to the topographical site of the *peripheral nervous system [C47]*, as these tumors are considered to arise from nerves [14]. *Fibromas*, *NOS [8810/0]* and *fibrosarcomas*, *NOS [8810/3]* diagnosed in the skin were coded to the appropriate topographical site of *soft tissues [C49]* [36].

The data included 585 male neutered dogs with testicular tumors and 59 female neutered dogs with ovarian tumors. As neutering is usually performed along with tumor removal at these sites, these dogs were considered intact at the time of tumor diagnosis. *Perianal skin [C21*.*3]*, *skin of vulva [C51*.*9]*, *skin of penis [C60*.*9]*, *and skin of scrotum*, *NOS [C63*.*2]* were reclassified to the topographical group of *skin [C44]* for analyses on tumor topography. We found nine tumors in 20-year-old or older dogs. Since the age information for these tumor cases could not be verified, they were excluded from the dataset.

#### Handling of multiple samples and multiple tumors from one individual

To identify samples from the same animal, records in the tumor dataset were sorted alphabetically both by owner name and address and screened manually for similarities in owner name, address, animal name, breed, and date of birth. For dogs with unknown date of birth, the approximate date of birth was calculated from the "date of diagnosis" and "age at diagnosis".

Multiple tumors in one individual were managed and handled according to the ICD-O-3 coding guidelines [37]. *Mast cell neoplasms [974]* simultaneously occurring in more than one topographical location in the *skin [C44]* or *subcutis [C49]*, neoplasms associated with the *mammary gland [C50]*, and benign tumors of the same type occurring in different sites were counted as separate cases. Tumors identified as a relapse (same tumor at the same site at a later time) were excluded from the data set. Only primary tumors were considered. For *hemangiosarcomas*, *NOS [9120/3]*, *lymphomas [*out of *959–972]*, *plasma cell myelomas [9732/3]*, *systemic mastocytosis*, *NOS [9741*.*1/3]*, and *histiocytic sarcomas [9755/3]*, the de-novo code *multiple sites [C80*.*91]* was assigned if the tumor was found in more than one topographical site and the available topographical codes were not applicable.

### Calculation of tumor incidence rates

An IR is defined as the ratio between the number of new disease cases and the total time of a population being exposed to the risk of disease [38]. The total number of years of dogs being exposed to the risk of developing a tumor during the study period constituted the dog-years at risk (DYAR). We calculated the IRs as the tumor rate per 100’000 DYAR using the following formula, slightly modified from a previous study [4]:

$$IR=\frac{N\;tumors*100’000}{(N\;dogs-N\;tumors)*mean\;DYAR\;for\;N\;dogs+N\;tumors*mean\;DYAR\;for\;N\;tumors}$$

The overall tumor IR as well as the IRs for benign and malignant tumors were calculated considering the respective numbers of tumor cases (*N tumors*) and of dogs registered in Switzerland (*N dogs*). IRs for specific age, sex, breeds, and tumor groups were calculated correspondingly. Breed-specific IRs were calculated for dog breeds with ≥200 individuals registered in Switzerland between 2008 and 2020.

For each dog in the reference dataset provided by Identitas AG, the effective DYAR during the observation period were calculated. Dogs that died on January 1^st^ were only considered at risk for the previous year(s). Because deregistration of dogs is not always reliably performed by dog owners, a data-based cutoff of 16 years was applied as the maximum life expectancy for dogs without a stated date of death. For each dog included in the tumor data set, DYAR during the observed timeframe were calculated based on date of birth and date of diagnosis, where possible, allowing calculation of precise DYAR for each individual and mean DYAR for each group analyzed.

Poisson 95 percent confidence intervals (95%CI) for IRs were generated with the *PoissonCI(x*, *n*, *method = c("exact"))* function from the R package “DescTools” version 0.99.48 [39], while *x* was the number of tumors and *n* was the DYAR of the reference population [40]. The results for lower and upper 95%CI were multiplied by 100’000 and thereby generated values were considered as 95%CI for IRs.

### Calculation of incidence rate ratios

IRRs of malignant tumors were assessed in a negative binomial regression analysis using the *glm*.*nb()* function included in the R package “MASS” [41], after tumors of *uncertain behavior [/1]* and *in situ neoplasms [/2]* were grouped as benign or malignant neoplasms according to S2 Table. The model was applied to a dataset containing aggregated data from both the SCCR dataset and the Swiss dog population dataset provided by Identitas AG. Cases from the tumor dataset and dogs from the Identitas AG dataset with missing information on one or more variables were omitted with *na*.*omit(df)*. For each possible combination of age, sex, and breed group and section (according to the Fédération Cynologique Internationale (FCI) [42] and national kennel clubs, as of March 2023), the number of malignant tumors along with DYAR from the Swiss dog population was indicated. A total of 21’803 malignant tumors were included in the negative binomial regression, with the dependent variable *tumors* (number of tumors) and the independent variables *age group* (0–3, 4–7, 8–11, 12–15, 16–19), *sex* (male/female), and *breed group and section* (mixed breed was an extra group). The underlying model was *glm*.*nb(tumors ~ age group + sex + breed group and section*, *offset(log(DYAR)))*. Age group 0–3, females, and mixed breeds were defined as references for the respective independent variables. IRRs and 95%CI were obtained using the *tbl_regression(model*, *exponentiate = TRUE)* function included in the R package “gtsummary” [43]. We considered results to be statistically significant if the 95%CI did not include or cross the value one.

## Results

Between January 2008 and December 2020, diagnoses were available from 128’247 canine samples submitted to four diagnostic laboratories. From these samples, 57’773 tumors were diagnosed, of which 54’986 tumors originated from 42’743 dogs located in Switzerland. A total of 41’363 tumors (75.22%) were diagnosed by histological analysis (40’023 (72.78%) biopsy samples and 1’340 (2.44%) necropsy samples) and 13’623 (24.77%) by cytological examination. The number of primary tumors diagnosed per dog ranged from one to 15.

### Tumor types and topographical location

Tumor categories recorded within the evaluated 13-year period are shown in Table 1. A total of 26’219 tumors (47.68%) were labeled with behavior code *benign [/0]*, 10’833 tumors (19.70%) were labeled as *uncertain whether benign or malignant [/1]*, 188 tumors (0.35%) were assigned with the code for *non-invasive or in situ neoplasms [/2]*, and 17’746 tumors (32.27%) were coded as *malignant [/3]*. After reassignment of *[/1]* and *[/2]* tumors, a total of 29’179 neoplasms were classified as benign and 24’076 tumors were classified as malignant. The five most frequent tumor categories according to the Vet-ICD-O-canine-1 classification included *lipomatous neoplasms [885–888]* (n = 7’954; 14.47%), *mast cell neoplasms [974]* (n = 5’485; 9.98%), *adnexal and skin appendage neoplasms [839–842]* (n = 4’421; 8.04%), *complex mixed and stromal neoplasms [893–899]* (n = 4’416; 8.03%), and *adenomas and adenocarcinomas [814–838]* (n = 4’146; 7.54%). The top five topographical tumor sites comprised the *skin [C44]* (n = 19’045; 34.64%), *soft tissues [C49]* (n = 11’092; 20.17%), the *mammary gland [C50]* (n = 7’974; 14.50%), *unknown sites [C80]* (n = 4’795; 8.72%), and *lymph nodes [C77]* (1’555; 2.83%).

**Table 1:** Tumor categories with absolute numbers, incidence rates per 100’000 dog-years at risk with 95% confidence intervals for all tumors, benign and malignant tumors, and relative distribution of diagnostic method for the 54’986 neoplasms registered in the Swiss Canine Cancer Registry, 2008–2020.

Tumor category [code]	N tumors	IR all tumors (95%CI)	IR benign tumors (95%CI)	IR malignant tumors (95%CI)	H (%)	C (%)
Neoplasms, NOS [800]	1’774	24.9 (23.7–26.0)	0.6 (0.4–0.8)	11.1 (10.3–11.9)	47.5	52.5
Epithelial neoplasms, NOS [801–804]	1’430	20.1 (19.0–21.1)	-	19.0 (18.0–20.0)	70.1	29.9
Squamous cell neoplasms [805–808]	2’704	37.9 (36.5–39.4)	23.4 (22.3–24.5)	14.5 (13.6–15.4)	97.8	2.2
Basal cell neoplasms [809–811]	2’306	32.3 (31.0–33.7)	31.4 (30.1–32.7)	0.9 (0.7–1.2)	95.1	4.9
Transitional cell papillomas and carcinomas [812–813]	270	3.8 (3.3–4.3)	-	3.8 (3.3–4.3)	71.1	28.9
Adenomas and adenocarcinomas [814–838]	4’146	58.2 (56.4–59.9)	20.6 (19.6–21.7)	37.4 (36.0–38.8)	92.9	7.1
Adnexal and skin appendage neoplasms [839–842]	4’421	62.0 (60.1–63.8)	49.8 (48.2–51.5)	8.8 (8.1–9.5)	85.2	14.8
Cystic, mucinous and serous neoplasms [844–849]	142	2.0 (1.7–2.3)	1.1 (0.9–1.4)	0.9 (0.7–1.1)	99.3	0.7
Ductal and lobular neoplasms [850–854]	200	2.8 (2.4–3.2)	1.3 (1.1–1.6)	1.5 (1.2–1.8)	100.0	0.0
Acinar cell neoplasms [855]	15	0.2 (0.1–0.3)	-	0.2 (0.1–0.3)	100.0	0.0
Thymic epithelial neoplasms [858]	42	0.6 (0.4–0.8)	-	0.6 (0.4–0.8)	81.0	19.0
Specialized gonadal neoplasms [859–867]	1’091	15.3 (14.4–16.2)	14.0 (13.1–14.9)	1.3 (1.1–1.6)	99.1	0.9
Paragangliomas and glomus tumors [868–871]	78	1.1 (0.9–1.4)	0.2 (0.1–0.4)	0.9 (0.7–1.1)	98.7	1.3
Melanocytomas and Melanomas [872–879]	2’897	40.6 (39.1–42.1)	15.0 (14.1–15.9)	24.5 (23.3–25.6)	93.3	6.7
Soft tissue tumors and sarcomas, NOS [880]	1’769	24.8 (23.7–26.0)	0.01 (0.00–0.08)	24.4 (23.3–25.6)	59.1	40.9
Fibromatous neoplasms [881–883]	2’045	28.7 (27.4–29.9)	5.0 (4.4–5.5)	23.7 (22.6–24.9)	99.8	0.2
Myxomatous neoplasms [884]	149	2.1 (1.8–2.5)	0.2 (0.1–0.3)	1.9 (1.6–2.2)	98.7	1.3
Lipomatous neoplasms [885–888]	7’954	111.6 (109.0–114.0)	110.1 (107.6–112.4)	1.5 (1.2–1.8)	27.5	72.5
Myomatous neoplasms [889–892]	335	4.7 (4.2–5.2)	3.3 (2.9–3.8)	1.4 (1.1–1.7)	99.7	0.3
Complex mixed and stromal neoplasms [893–899]	4’416	61.9 (60.1–63.7)	41.6 (40.1–43.1)	15.8 (14.9–16.7)	99.5	0.5
Fibroepithelial neoplasms [900–903]	20	0.3 (0.2–0.4)	0.3 (0.2–0.4)	-	100.0	0.0
Synovial-like neoplasms [904]	15	0.2 (0.1–0.3)	-	0.2 (0.1–0.3)	100.0	0.0
Mesothelial neoplasms [905]	28	0.4 (0.3–0.6)	-	0.4 (0.3–0.6)	92.9	7.1
Germ cell neoplasms [906–909]	510	7.2 (6.5–7.8)	7.0 (6.4–7.6)	0.1 (0.1–0.2)	99.8	0.2
Blood vessel tumors [912–916]	2’325	32.6 (31.3–33.9)	14.5 (13.6–15.4)	18.1 (17.1–19.1)	100.0	0.0
Lymphatic vessel tumors [917]	4	0.1 (0.0–0.1)	0.0 (0.0–0.1)	0.0 (0.0–0.1)	100.0	0.00
Osseous and chondromatous neoplasms [918–924]	566	7.9 (7.3–8.6)	0.4 (0.2–0.5)	7.6 (6.9–8.2)	95.1	4.9
Miscellaneous bone tumors [926]	1	0.0 (0.0–0.1)	0.0 (0.0–0.1)	-	100.0	0.0
Odontogenic tumors [927–934]	534	7.5 (6.9–8.1)	7.5 (6.8–8.1)	0.0 (0.0–0.1)	100.0	0.0
Miscellaneous tumors [935–937]	10	0.1 (0.1–0.3)	0.1 (0.1–0.3)	-	60.0	40.0
Gliomas [938–948]	145	2.0 (1.7–2.4)	0.1 (0.0–0.2)	1.9 (1.6–2.2)	100.0	0.0
Neuroepitheliomatous neoplasms [949–952]	9	0.1 (0.1–0.2)	-	0.1 (0.0–0.2)	100.0	0.0
Meningiomas [953]	87	1.2 (1.0–1.5)	0.7 (0.5–0.9)	0.2 (0.1–0.4)	100.0	0.0
Nerve sheath tumors [954–957]	67	0.9 (0.7–1.2)	0.4 (0.2–0.5)	0.6 (0.4–0.8)	100.0	0.0
Granular cell tumors [958]	10	0.1 (0.1–0.3)	0.1 (0.1–0.2)	0.0 (0.0–0.1)	100.0	0.0
Malignant lymphomas, NOS or diffuse [959–972]	2’585	36.3 (34.8–37.7)	-	36.3 (34.8–37.7)	33.2	66.8
Plasma cell neoplasms [973]	620	8.7 (8.0–9.4)	8.4 (7.7–9.1)	0.3 (0.2–0.5)	85.2	14.8
Mast cell neoplasms [974]	5’485	77.0 (74.9–78.9)	6.6 (6.0–7.2)	70.4 (68.4–72.3)	74.6	25.4
Neoplasms of histiocytes and accessory lymphoid cells [975]	3’740	52.5 (50.7–54.1)	45.6 (44.0–47.1)	6.9 (6.3–7.5)	71.7	28.3
Immunoproliferative diseases [976]	1	0.0 (0.0–0.1)	-	-	100.0	0.0
Lymphoid leukemias [981–983]	27	0.4 (0.2–0.6)	-	0.4 (0.2–0.6)	29.6	70.4
Myeloid leukemias [984–993]	4	0.1 (0.0–0.1)	-	0.1 (0.0–0.1)	75.0	25.0
Myeloproliferative neoplasms [995–996]	9	0.1 (0.1–0.2)	-	0.1 (0.1–0.2)	100.0	0.0
Grand Total	54’986	775.3 (764.2–777.1)	410.5 (404.3–413.7)	338.3 (333.2–341.7)	75.2	24.8

IR: incidence rate (tumors per 100‘000 dog-years at risk); N: number; 95%CI: 95% confidence interval; H: histology (biopsy and necropsy cases), C: cytology; NOS: not otherwise specified; -: no value.

### Age distribution

**Fig 1:**
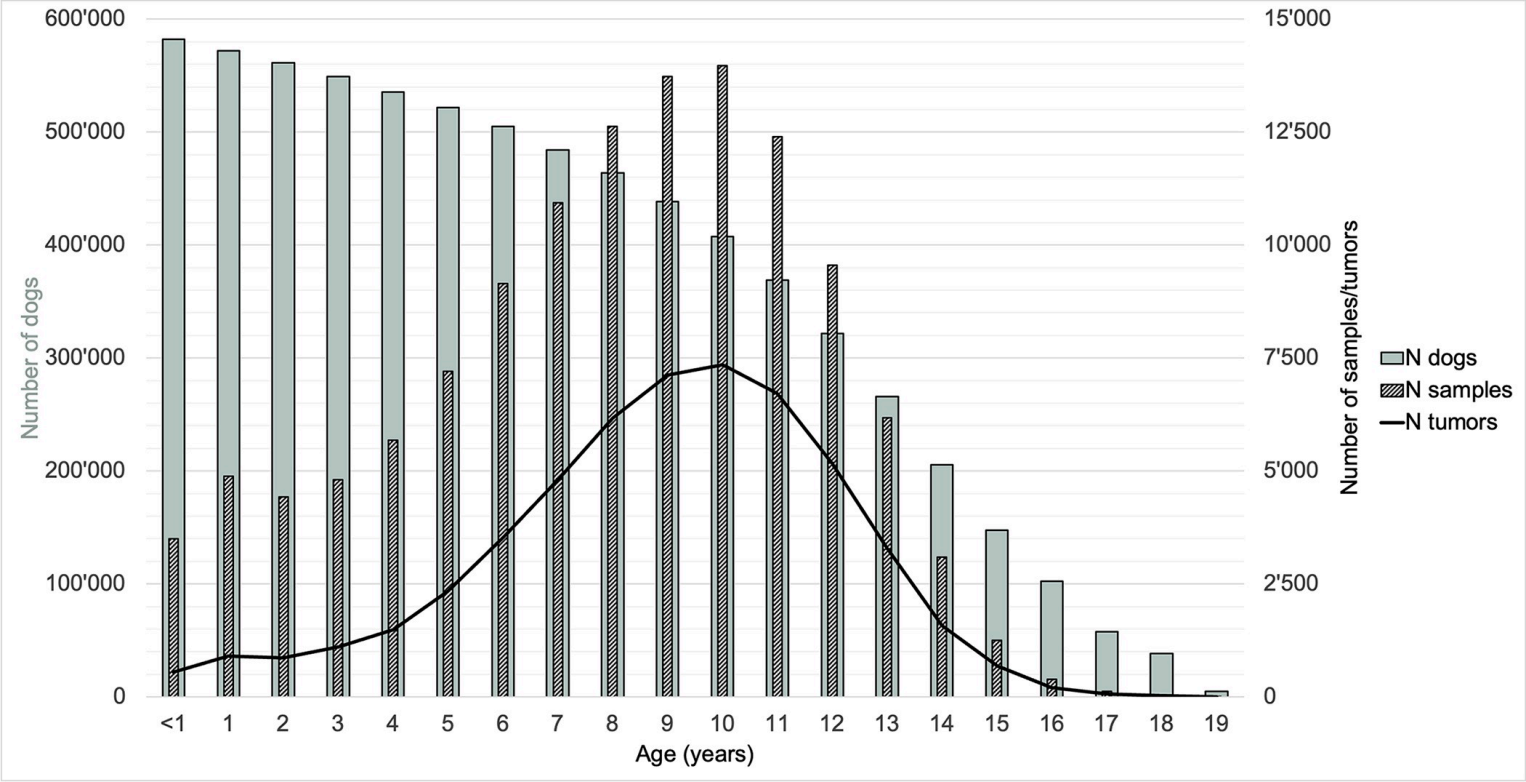
Number of dogs registered in the Swiss animal registration database Amicus, number of samples submitted, and number of tumors diagnosed in relation to age from 2008 to 2020. N: number.

The dataset included tumors of dogs from <1 to 19 years of age. The age at tumor diagnosis was available for 53’976 tumors. The median age was nine years, the mean age 8.96 years. Ten years was the age at which the number of tumors peaked (n = 7’354; 13.37%). One quarter of all tumors were diagnosed by the age of seven years (n = 15’569; 28.31%) and three quarters by the age of eleven years (n = 42’938; 78.09%). This distribution reflects approximately the age distribution of the total sample submission but differs from the age distribution of the whole dog population (Fig 1).

### Sex distribution

Most of the 54’986 tumors originated from female dogs (n = 30’540; 55.54%), 15’577 tumors (28.33%) thereof from neutered females and 14’963 tumors (27.21%) from intact females. In males (n = 23’733; 43.16%), most tumors were seen in intact dogs (n = 13’712; 24.94%), and the smallest number of tumors overall derived from male neutered dogs (n = 10’021; 18.22%). For 713 (1.30%) tumors, the sex of the dog was not reported. Sex distribution for the ten most common tumor groups is shown in Table 2.

**Table 2:** The ten most common tumor groups and the respective distribution of sex; the top five breeds with the highest incidence rates, and age range at increased risk.

Tumor group [code]	N tumors and age range at increased risk^1^	Sex distribution (%)	Dog breed with highest IR	IR (95%CI)
Lipomas [out of 885–888]	7‘847	MN (26.42)	Doberman Pinscher	604 (481–745)
		M (18.07)	Polski Owczarek Nizinny	554 (305–900)
		FN (34.56)	Magyar Vizsla	547 (442–668)
		F (19.75)	Flat Coated Retriever	535 (466–610)
		Unk (1.20)	Cão de água português	427 (223–755)
	7–12 years		Average all breeds	110 (108–112)
Mast cell tumors [974]	5‘485	MN (17.37)	Dogo Argentino	844 (591–1‘169)
		M (23.90)	Boxer	731 (654–800)
		FN (34.59)	Nova Scotia Duck Tolling Retriever	697 (549–875)
		F (22.72)	Rhodesian Ridgeback	531 (443–628)
		Unk (1.42)	Manchester Terrier	479 (208–951)
	7–12 years		Average all breeds	77 (75–79)
Canine cutaneous histiocytomas [9751.1/0]	3‘247	MN (19.68)	Flat Coated Retriever	617 (535–689)
		M (37.36)	Boxer	316 (267–364)
		FN (18.76)	Russian Black Terrier	296 (108–641)
		F (22.36)	Dogo Canario	274 (74–697)
		Unk (1.85)	Doberman Pinscher	257 (179–353)
	1–2 years		Average all breeds	46 (44–47)
Complex mixed and stromal adenomas [out of 893–889]	2‘964	MN (0.24)	Field Spaniel	1‘528 (962–2‘235)
		M (0.47)	Russian Black Terrier	486 (235–902)
		FN (25.74)	Welsh Springer Spaniel	265 (107–548)
		F (72.33)	Saluki	227 (98–448)
		Unk (1.21)	Koojkerhondje	226 (132–363)
	7–12 years		Average all breeds	42 (40–43)
Adnexal and skin appendage adenomas [out of 839–842]	2‘837	MN (22.70)	Schnauzer—Standard	361 (254–498)
		M (37.40)	Lakeland Terrier	233 (85–505)
		FN (26.51)	Polski Owczarek Nizinny	218 (80–475)
		F (12.09)	Bobtail (Old English Sheepdog)	190 (109–308)
		Unk (1.30)	Afghan Hound	179 (77–352)
	8–14 years		Average all breeds	40 (38–41)
Adenocarcinomas [out of 814–838]	2‘669	MN (14.05)	Field Spaniel	376 (138–817)
		M (14.54)	Polski Owczarek Nizinny	182 (59–424)
		FN (30.76)	Gordon Setter	165 (98–261)
		F (39.34)	Scottish Terrier	161 (80–288)
		Unk (1.31)	Welsh Springer Spaniel	151 (41–389)
	8–14 years		Average all breeds	37 (36–39)
Lymphomas [959–972]	2‘585	MN (21.66)	King Charles Spaniel	319 (137–627)
		M (29.86)	Manchester Terrier	302 (98–704)
		FN (28.74)	Dogue de Bordeaux	275 (146–469)
		F (18.53)	Polski Owczarek Nizinny	257 (102–525)
		Unk (1.20)	Airedale Terrier	186 (113–287)
	8–13 years		Average all breeds	36 (39–42)
Basal cell neoplasms, benign [out of 809–881]	2‘241	MN (18.65)	Polski Owczarek Nizinny	624 (360–990)
		M (25.08)	Irish Soft Coated Wheaten Terrier	493 (320–718)
		FN (31.64)	Gordon Setter	413 (301–552)
		F (23.29)	Russian Black Terrier	345 (138–708)
		Unk (1.34)	Briard	302 (198–438)
	7–11 years		Average all breeds	31 (30–33)
Melanomas [out of 872–879]	1‘744	MN (22.19)	Russian Black Terrier	345 (138–708)
		M (31.19)	Schnauzer—Giant	286 (209–381)
		FN (25.11)	Magyar Vizsla	266 (194–353)
		F (20.18)	Airedale Terrier	261 (173–376)
		Unk (1.32)	Irish Terrier	200 (100–357)
	9–15 years		Average all breeds	24 (23–26)
Sarcomas [out of 880]	1‘743	MN (22.78)	Bullmastiff	151 (49–352)
		M (23.35)	Borzoi	132 (48–287)
		FN (30.41)	Rottweiler	107 (73–150)
		F (21.74)	Boxer	101 (76–131)
		Unk (1.72)	Flat Coated Retriever	94 (66–128)
	8–14 years		Average all breeds	24 (23–26)

IR: incidence rate (tumors per 100‘000 dog-years at risk); N: number; 95%CI: 95% confidence interval; MN: male neutered, M: male intact, FN: female neutered, F: female intact, Unk: unknown; ^1^The age range in which age-specific IRs are higher than 1.5 times the average IR of all breeds for the respective tumor group.

### Breed distribution

The dataset (n = 54’986 tumors/n = 42’743 dogs) comprised 368 different breed categories: 325 defined dog breeds, 41 NOS categories (e.g., Basset NOS, Poodle NOS, Schnauzer NOS, Spitz NOS), the category of mixed breed, and one category of unknown breed. Among the 42’743 individual dogs, mixed breeds were the most common (n = 9’916; 23.20%), followed by Labrador Retrievers (n = 2’844; 6.65%), Golden Retrievers (n = 1’897; 4.44%), and German Shepherds (n = 1’052; 2.46%). The breed was unknown for 2’702 (6.32%) dogs. Most of the 54’986 tumor samples originated from mixed breed dogs (n = 12’389; 22.53%), followed by Labrador Retrievers (n = 3’706; 6.74%), Golden Retrievers (n = 2’468; 4.49%), and Boxers (n = 1’416; 2.58%). The breed was unknown for 3’133 (5.70%) tumor diagnoses. The number of tumor samples recorded in the SCCR for the 20 most common Swiss dog breeds between 2008 and 2020 is shown in Table 3.

**Table 3:** The 20 most popular Swiss dog breeds registered in Amicus and the corresponding tumor data from the Swiss Canine Cancer Registry, from 2008 to 2020.

Dog breed	N dogs Amicus	DYAR Amicus	N tumors (%)	DYAR tumors	IR all tumors (95%CI)	IR benign tumors (95%CI)	IR malignant tumors (95%CI)
Mixed breed	296’059	2’142’321	12’389 (22.53)	78’913	581 (568–589)	304 (295–310)	259 (251–265)
Labrador Retriever	49’357	342’826	3’706 (6.74)	23’852	1’087 (1’046–1’116)	586 (559–610)	475 (451–497)
Chihuahua	36’496	268’033	434 (0.79)	3’543	162 (147–178)	100 (89–113)	55 (47–65)
Yorkshire Terrier	35’136	256’158	1’132 (2.06)	7’440	443 (417–468)	272 (252–292)	148 (133–163)
Jack Russell Terrier	28’613	223’769	1’153 (2.10)	7’669	518 (486–546)	271 (249–293)	228 (209–249)
Golden Retriever	28’210	200’238	2’468 (4.49)	14’480	1’252 (1’184–1’282)	568 (532–598)	646 (607–677)
German Shepherd	27’051	172’420	1’293 (2.35)	7’570	753 (710–792)	372 (343–401)	360 (332–390)
Border Collie	23’729	160’368	626 (1.14)	4’416	390 (360–422)	194 (173–217)	185 (165–208)
Poodle (1)	22’330	158’402	1’103 (2.01)	7’372	698 (656–739)	455 (421–488)	221 (198–245)
Bernese Mountain Dog	19’362	114’247	1’227 (2.23)	7’092	1’075 (1’015–1’136)	408 (372–447)	633 (588–681)
French Bulldog	19’017	108’921	767 (1.39)	4’738	702 (655–756)	380 (343–418)	300 (270–337)
Belgian Shepherd (2)	15’105	103’899	782 (1.42)	5’082	755 (701–807)	359 (323–396)	368 (332–406)
Dachshund (3)	14’134	96’939	800 (1.45)	4’961	830 (769–884)	521 (475–566)	275 (242–309)
Cocker Spaniel (4)	14’116	101’137	1’352 (2.46)	8’683	1’350 (1’266–1’410)	791 (732–843)	499 (455–543)
Spitz (5)	13’547	68’899	154 (0.28)	1’104	222 (190–262)	135 (109–165)	75 (56–99)
West Highland White Terrier	11’396	86’812	693 (1.26)	3’972	812 (740–860)	429 (383–471)	338 (299–377)
Maltese	10’831	73’227	251 (0.46)	1’857	342 (302–388)	179 (150–212)	155 (128–187)
Husky (6)	10’558	71’336	377 (0.69)	2’470	529 (476–585)	257 (221–297)	251 (216–290)
Appenzell Cattle Dog	10’390	64’971	220 (0.40)	1’321	339 (295–386)	148 (120–180)	179 (148–214)
Shih Tzu	9’287	70’077	254 (0.46)	1’644	364 (319–410)	219 (185–256)	123 (98–152)
Grand Total	1’032’029	7’135’182	54’986 (100)	340’513	775 (764–777)	410 (404–414)	338 (333–342)

IR: incidence rate (tumors per 100‘000 dog-years at risk); N: number; 95%CI: 95% confidence interval; DYAR: dog-years at risk; (1) includes: Standard Poodle, Medium Poodle, Miniature Poodle, Toy Poodle, Poodle NOS; (2) includes: Groenendael, Laekenois, Malinois, Tervueren, Belgian Shepherd NOS; (3) includes: Rabbit Dachshund, Miniature Dachshund, Dachshund long-haired, Dachshund short-haired, Dachshund wire-haired, Dachshund NOS; (4) includes: American Cocker Spaniel, English Cocker Spaniel, Cocker Spaniel NOS; (5) includes: German Spitz, Giant Spitz, Medium Spitz, Miniature Spitz, Pomeranian, Wolfspitz, Japan Spitz, Spitz NOS; (6) includes: Siberian Husky, Alaskan Husky, Husky NOS; NOS: not otherwise specified.

### Tumor incidence rates

**Fig 2:**
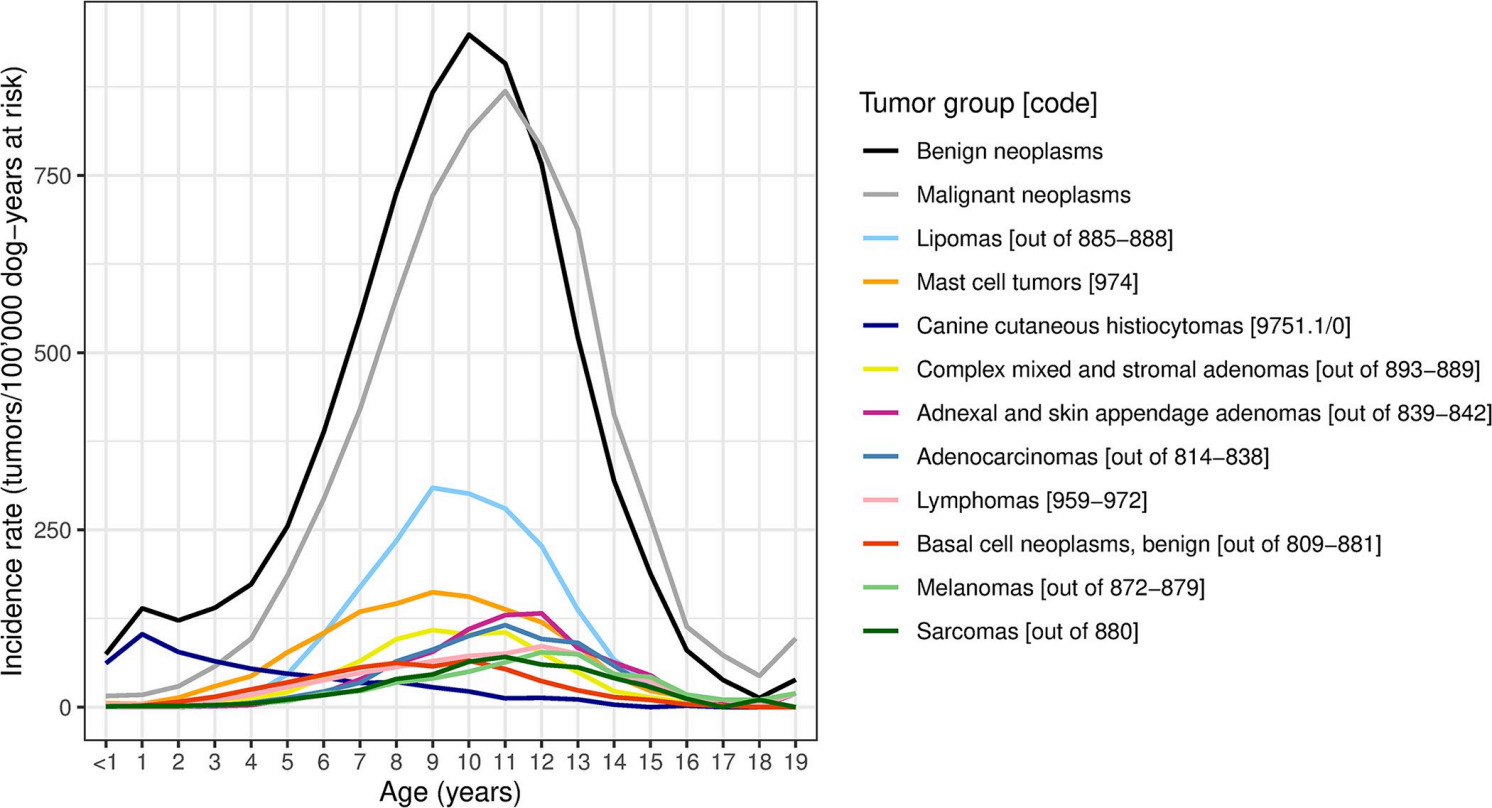
Incidence rates of the ten most common tumor groups and overall tumor incidence rates for benign and malignant neoplasms in relation to age.

All dogs registered in Switzerland between 2008 and 2020 contributed for 7’135’182 dog-years (3’619’335 female dog-years; 3’515’847 male dog-years). The mean DYAR between 2008 and 2020 was 6.91 for all dogs (6.98 for female dogs and 6.84 for male dogs). The number of 54’986 reported tumors resulted in 340’513 DYAR (190’017 female DYAR; 146’154 male DYAR; and 4’342 DYAR of unknown sex), with a mean DYAR of 6.19 until tumor diagnosis for all dogs with tumors, 6.22 DYAR for females and 6.16 DYAR for males. The overall tumor IR was 775 tumors/100’000 DYAR (95%CI 764–777). For female dogs (intact and neutered) the IR was 850 (95%CI 834–853), for male dogs (intact and neutered) the IR was 679 (95%CI 666–684). Dogs between seven and 13 years of age had an IR higher than the average IR, with the highest IR at eleven years of age with 1’857 tumors/100’000 dogs per year (95%CI 1’780–1’867). After grouping tumors according to S2 Table, we found that benign tumors (IR: 410; 95%CI 404–414) peaked at the age of ten years (IR: 948; 95%CI 910–970) and malignant tumors (IR: 338; 95%CI 333–342) at the age of eleven years (IR: 869; 95%CI 832–892; Fig 2). The IRs for the 20 most common Swiss dog breeds between 2008 and 2020 are listed in Table 3.

### Breed-specific tumor incidence rates

Dog breeds (precise breed) with at least 200 individuals registered in Switzerland between 2008 and 2020 were considered. We found the highest tumor IR in the Polski Owczarek Nizinny (PON) (IR: 3’303; 95%CI 2’502–3’864), followed by the Magyar Vizsla (IR: 3’154; 95%CI 2’824–3’352) and the Flat Coated Retriever (FCR) (IR: 3’024; 95%CI 2’778–3’114). The 5 breeds with the highest overall tumor IRs also included Giant Schnauzers (IR: 2’920; 95%CI 2’632–3’164) and Doberman Pinschers (IR: 2’865; 95%CI 2’530–3’090). In these breeds, the IRs for benign tumors were higher than the IRs for malignant tumors, with the exception of the Giant Schnauzer (Table 4). Interestingly, 18 and 17 of the 20 breeds with the highest overall tumor IRs were also among the 20 breeds with the highest IRs for benign and malignant tumors, respectively (S8 and S9 Tables).

**Table 4:** The 20 Swiss dog breeds (precise breed) with the highest overall tumor incidence rates between 2008 and 2020 and their respective Swiss Canine Cancer Registry data.

Dog breed (precise)	N dogs Amicus	DYAR Amicus	N tumors (%)	DYAR tumors	IR all tumors (95%CI)	IR benign tumors (95%CI)	IR malignant tumors (95%CI)
Polski Owczarek Nizinny	369	2’749	86 (0.16)	495	3’303 (2’502–3’864)	2’349 (1’729–2’891)	846 (530–1’255)
Magyar Vizsla	2’419	17’373	535 (0.97)	3’431	3’154 (2’824–3’352)	1’896 (1’667–2’079)	1’137 (976–1’298)
Flat Coated Retriever	6’169	40’615	1’195 (2.17)	6’773	3’024 (2’778–3’114)	1’689 (1’534–1’787)	1’164 (1’053–1’264)
Schnauzer—Giant	2’423	16’097	465 (0.85)	2’918	2’920 (2’632–3’164)	1’352 (1’169–1’533)	1’466 (1’279–1’659)
Doberman Pinscher	2’452	14’110	395 (0.72)	1’951	2’865 (2’530–3’090)	1’546 (1’327–1’742)	1’177 (998–1’362)
Schnauzer—Standard	1’405	10’244	279 (0.51)	1’772	2’795 (2’413–3’063)	1’445 (1’203–1’676)	1’285 (1’060–1’507)
Field Spaniel	201	1’598	44 (0.08)	327	2’793 (2’001–3’696)	1’969 (1’318–2’754)	813 (433–1’391)
Rhodesian Ridgeback	3’772	24’922	678 (1.23)	4’396	2’730 (2’520–2’933)	1’416 (1’269–1’568)	1’278 (1’140–1’424)
Boxer	8’336	53’684	1’416 (2.58)	7’634	2’713 (2’502–2’779)	1’182 (1’075–1’259)	1’442 (1’326–1’530)
Black Russian Terrier	324	2’038	54 (0.10)	317	2’679 (1’991–3’457)	1’712 (1’196–2’388)	695 (376–1’153)
Airedale Terrier	1’556	10’772	279 (0.51)	1’679	2’652 (2’295–2’912)	1’601 (1’350–1’834)	983 (797–1’180)
Nova Scotia Duck Tolling Retriever	1’636	10’744	287 (0.52)	2’001	2’643 (2’371–2’999)	1’357 (1’156–1’608)	1’245 (1’045–1’477)
Gordon Setter	1’570	10’900	280 (0.51)	1’736	2’619 (2’277–2’888)	1’435 (1’207–1’664)	1’065 (871–1’266)
Irish Terrier	765	5’512	133 (0.24)	882	2’447 (2’020–2’860)	1’394 (1’086–1’726)	965 (720–1’258)
Bouvier des Flandres	367	2’364	51 (0.09)	229	2’252 (1’606–2’837)	988 (617–1’460)	1’171 (753–1’662)
Dogo Argentino	712	4’265	95 (0.17)	549	2’238 (1’802–2’723)	1’132 (830–1’492)	1’076 (790–1’439)
King Charles Spaniel	406	2’516	48 (0.09)	264	1’934 (1’407–2’529)	1’006 (643–1’467)	875 (548–1’324)
Briard	1’363	8’971	164 (0.30)	977	1’849 (1’559–2’130)	1’056 (847–1’282)	740 (569–936)
Irish Soft Coated Wheaten Terrier	668	5’308	90 (0.16)	629	1’724 (1’363–2’084)	1’457 (1’128–1’792)	263 (144–443)
Dogue de Bordeaux	796	4’736	80 (0.15)	447	1’700 (1’339–2’102)	743 (515–1’028)	795 (639–1’199)
Grand Total	1’032’029	7’135’182	54’986 (100.00)	340’513	775 (764–777)	410 (404–414)	338 (333–342)

IR: incidence rate (tumors per 100‘000 dog-years at risk); N: number; 95%CI: 95% confidence interval; DYAR: dog-years at risk.

### The ten most frequent tumor groups: IRs and distribution for age, sex, and topography

**Fig 3:**
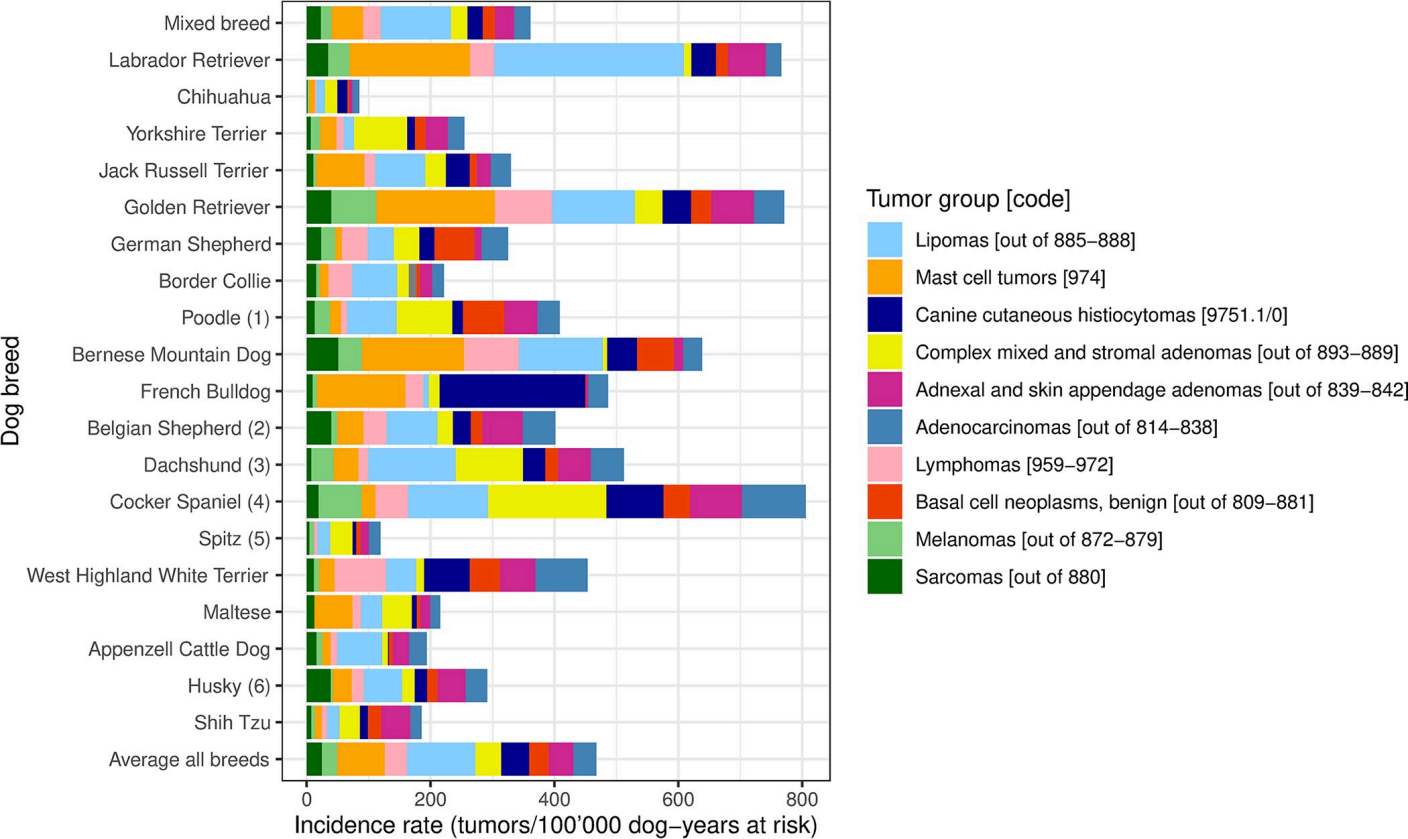
Incidence rates of the ten most common tumor groups in the 20 most popular Swiss dog breeds. (1) includes: Standard Poodle, Medium Poodle, Miniature Poodle, Toy Poodle, Poodle NOS; (2) includes: Groenendael, Laekenois, Malinois, Tervueren, Belgian Shepherd NOS; (3) includes: Rabbit Dachshund, Miniature Dachshund, Dachshund long-haired, Dachshund short-haired, Dachshund wire-haired, Dachshund NOS; (4) includes: American Cocker Spaniel, English Cocker Spaniel, Cocker Spaniel NOS; (5) includes: German Spitz, Giant Spitz, Medium Spitz, Miniature Spitz, Pomeranian, Wolfspitz, Japan Spitz, Spitz NOS; (6) includes: Siberian Husky, Alaskan Husky, Husky NOS; NOS: not otherwise specified.

Groups of neoplasms were established along the morphological classification indicated by Vet-ICD-O-canine-1 and according to biological behavior. *Mast cell neoplasms [974]* were grouped together regardless of behavior. The IRs for the ten most common tumor groups in the 20 most popular Swiss dog breeds are displayed in Fig 3. Breeds that were not diagnosed with any tumor of the ten most common tumor groups or with any other tumor are listed in S3 Table. Table 2 shows the ten most common tumor groups, the age range with increased IRs (age range in which age-specific IRs are higher than 1.5 times the average IR of all breeds for the respective tumor group), the relative frequencies of sex, and the dog breeds (breeds with ≥200 individuals registered in Switzerland between 2008 and 2020) with the respective highest IRs. The breeds with the highest tumor IRs for a particular group of tumors had four to 36-fold increased IRs compared to the average IR for all breeds.

### Other frequently diagnosed tumors

**Fig 4:**
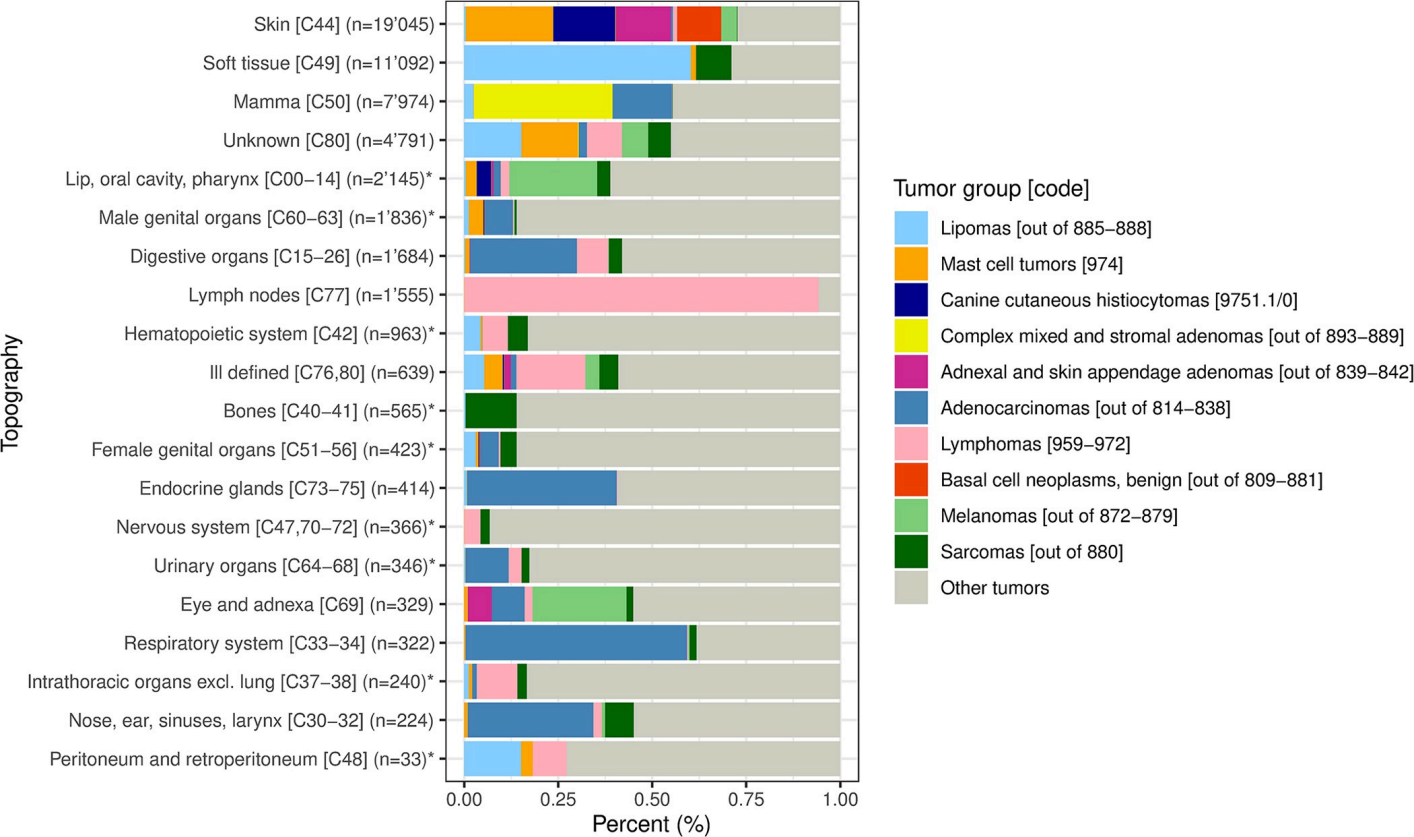
Relative frequency (%) of the ten most common tumor groups and the group of “other tumors” per topographical location. *Topographical locations where “other tumors” make up more than 60% of all tumors.

The relative distribution of the ten most diagnosed tumor groups and “other tumors” per topographical location is shown in Fig 4. “Other tumors” accounted for more than 60% of all tumors in nine topographical sites (S4 Table). The distribution of tumor categories over these nine topographical sites is shown in S5 Table. In the *lip*, *oral cavity and pharynx [C00-14]*, *melanocytomas and melanomas [872–879]* were the most represented tumor category. *Male genital organs [C60-63]* were frequently affected by *specialized gonadal neoplasms [859–867]*, while in the *hematopoietic system [C42]* and in *intrathoracic organs (excl*. *lung) [C37-38] blood vessel tumors [912–916]* were most common. In *bones [C40-41]*, *osseous and chondromatous neoplasms [918–924]* were most often diagnosed and in *female genital organs [C51-56] myomatous neoplasms [889–892]* were most frequent. The largest tumor category in the *urinary organs [C64-68]* consisted of *transitional cell papillomas and carcinomas [812–813]* and in the *nervous system [C47*,*70–72]* of *gliomas [938–948]*, respectively. Most neoplasms in the *peritoneum and retroperitoneum [C48]* belonged to the category of *epithelial neoplasms*, *NOS [801–804]*. *Blood vessel tumors [912–916]* frequently occurred in the *soft tissue [C49]*, while most *melanomas and melanocytomas [872–879]* occurred in the *skin [C44]*. The quantitatively most represented tumor categories per each of the nine topographical locations along with the age range with increased IRs (age range in which age-specific IRs are higher than 1.5 times the average IR of all breeds for the respective tumor category), the breeds with the highest IRs for the respective category, and the IRs for benign and malignant tumors are presented in Table 5.

**Table 5:** Largest tumor categories from nine topographical sites where the group of”other tumors” accounted for more than 60% of all tumors and the top five breeds with the highest overall incidence rates, incidence rates for benign and malignant tumors, and age range at increased risk.

Tumor category [code]	N tumors and age range at increased risk^1^	Dog breed with highest overall tumor IR	Overall tumor IR (95%CI)	IR benign tumors (95%CI)	IR malignant tumors (95%CI)
Blood vessel tumors [912–916]	2‘325	Dogo Argentino	257 (129–461)	140 (52–306)	117 (38–274)
		Boxer	239 (199–283)	170 (136–208)	69 (49–95)
		Airedale Terrier	177 (106–275)	130 (71–218)	46 (15–108)
		Bouvier des Flandres	170 (46–433)	42 (1–236)	127 (26–371)
		Borzoi	154 (62–317)	66 (14–193)	88 (24–225)
	8–12 years	Average all breeds	33 (31–34)	15 (14–15)	18 (17–19)
Epithelial neoplasms, NOS [801–804]	1‘430	Afghan Hound	90 (24–229)	90 (24–229)	-
		Australian Silky Terrier	88 (18–255)	29 (1–162)	-
		Springer Spaniel	87 (48–146)	68 (34–122)	-
		King Charles Spaniel	80 (10–287)	40 (1–221)	-
		Doberman Pinscher	78 (39–139)	78 (39–139)	-
	8–14 years	Average all breeds	20 (19–21)	-	19 (18–20)
Specialized gonadal neoplasms [859–867]	1‘091	Lakeland Terrier	155 (42–396)	155 (42–396)	-
		Afghan Hound	134 (49–292)	112 (36–261)	22 (1–124)
		King Charles Spaniel	120 (25–348)	80 (10–287)	40 (1–221)
		Border Terrier	118 (72–182)	106 (63–167)	12 (1–43)
		Saluki	114 (31–291)	114 (31–291)	-
	9–14 years	Average all breeds	15 (14–16)	14 (13–15)	1 (1–2)
Melanocytomas [out of 872–879]	1‘071	Magyar Vizsla	404 (314–509)	404 (314–509)	-
		Nova Scotia Duck Tolling Retriever	372 (266–507)	372 (266–507)	-
		Australian Terrier	367 (158–721)	367 (158–721)	-
		Irish Terrier	290 (166–471)	290 (166–471)	-
		Rhodesian Ridgeback	289 (226–364)	289 (226–364)	-
	7–11 years	Average all breeds	15 (14–16)	15 (14–16)	-
Osseous and chondromatous neoplasms [918–924]	566	Deerhound	150 (18–541)	-	150 (18–541)
		Rottweiler	93 (62–135)	-	93 (62–135)
		Borzoi	88 (24–225)	-	88 (24–225)
		Irish Wolfhound	86 (18–251)	-	86 (18–251)
		Landseer	74 (15–216)	-	74 (15–216)
	7–12 years	Average all breeds	8 (7–9)	-	8 (7–8)
Myomatous neoplasms [889–892]	335	Welsh Terrier	49 (6–178)	25 (1–138)	25 (1–138)
		Irish Terrier	36 (4–131)	36 (4–131)	-
		Volpino Italiano	31 (4–113)	31 (4–113)	-
		Saluki	28 (1–158)	28 (1–158)	-
		Greyhound	28 (1–154)	-	28 (1–154)
	9–15 years	Average all breeds	5 (4–5)	3 (3–4)	1 (1–2)
Transitional cell papillomas and carcinomas [812–813]	270	Scottish Terrier	132 (60–250)	-	132 (60–250)
		Kuvasz	67 (2–373)	-	67 (2–373)
		Podenco Ibicenco	64 (2–356)	-	64 (2–356)
		Norfolk Terrier	35 (1–195)	-	35 (1–195)
		Bullmastiff	30 (1–168)	-	30 (1–168)
	9–13,15 years	Average all breeds	4 (3–4)	-	4 (3–4)
Gliomas [938–948]	145	Bearded Collie	91 (45–162)	-	91 (45–162)
		Cane Corso Italiano	34 (7–99)	-	34 (7–99)
		Boxer	32 (18–51)	2 (0–10)	30 (17–48)
		French Bulldog	31 (22–44)	-	30 (21–43)
		Bullmastiff	30 (1–168)	-	30 (1–168)
	7–9 years	Average all breeds	2 (2–2)	-	2 (2–2)

IR: incidence rate (tumors per 100‘000 dog-years at risk); N: number; 95%CI: 95% confidence interval; ^1^The age range in which age-specific IRs are higher than 1.5 times the average IR of all breeds for the respective tumor category, -: no value.

### Incidence rate ratios for malignant tumors

The IRRs for malignant tumors were assessed using a negative binomial regression model, adjusting for age groups, sex, and breed groups and sections (Table 6). The highest IRR compared to the 0–3 year age group was found in the 8–11 year age group (IRR: 18.2; CI95% 15.8–21.0). Compared to females, males had a lower IRR of being diagnosed with a malignant tumor (IRR: 0.74; 95%CI 0.69–0.80). The breed groups and sections with the highest IRR for developing a malignant tumor compared to mixed breeds were Nordic hunting dogs (Group 5, Section 2; IRR: 8.08; CI95% 3.55–16.7), rough-haired Sighthounds (Group 10, Section 2; IRR. 6.88; CI95% 4.03–11.5), and Cattledogs (Group 1, Section 2; IRR: 3.81; CI95% 2.43–5.88). The lowest IRRs among the different breed groups and sections for the development of malignant tumors compared to mixed breeds were found in Chihuahueno (Group 9, Section 6; IRR: 0.42; 95%CI 0.31–0.57), followed by toy Terriers (Group 3, Section 4; IRR: 0.68; 95%CI 0.53–0.88) and Bichons and related breeds (Group 9, Section 1; IRR: 0.73; CI95% 0.56–0.95).

**Table 6:** Results from the negative binomial regression model showing the incidence rate ratios of 21’803 malignant tumors in the Swiss Canine Cancer Registry (2008–2020) for age group, sex, and breed group and section.

Variable	Value	IRR	95%CI
Age group	0–3	—	—
	4–7	6.1	5.28–7.05
	8–11	18.2	15.8–21.0
	12–15	15.6	13.4–18.1
	16–19	4.04	3.08–5.29
Sex	Female	—	—
	Male	0.74	0.69–0.80
Breed group and section	Mixed breed	—	—
	Sheepdogs (Group 1, Section 1)	1.13	0.90–1.42
	Cattledogs (except Swiss Cattledogs) (Group 1, Section 2)	3.81	2.43–5.88
	Pinscher and Schnauzer type (Group 2, Section 1)	2.78	2.18–3.56
	Molossian type (Group 2, Section 2)	3.33	2.63–4.22
	Swiss Mountain- and Cattledogs (Group 2, Section 3)	2.14	1.69–2.73
	Large and medium sized Terriers (Group 3, Section 1)	1.57	1.21–2.03
	Small sized Terriers (Group 3, Section 2)	1.13	0.89–1.44
	Bull type Terriers (Group 3, Section 3)	2.42	1.85–3.18
	Toy Terriers (Group 3, Section 4)	0.68	0.53–0.88
	Dachshunds (Group 4)	1.21	0.92–1.60
	Nordic sledge dogs (Group 5, Section 1)	1.30	0.99–1.71
	Nordic hunting dogs (Group 5, Section 2)	8.08	3.55–16.7
	Nordic Watchdogs and Herders (Group 5, Section 3)	2.64	1.20–5.32
	European Spitz (Group 5, Section 4)	0.79	0.55–1.13
	Asian Spitz and related breeds (Group 5, Section 5)	2.12	1.54–2.91
	Primitive type (Group 5, Section 6)	3.68	1.95–6.63
	Primitive type—hunting dogs (Group 5, Section 7)	2.53	1.69–3.77
	Scent Hounds (Group 6, Section 1)	1.38	1.07–1.79
	Leash (scent) Hounds (Group 6, Section 2)	1.87	1.19–2.88
	Related breeds (Group 6, Section 3)	3.54	2.72–4.60
	Continental pointing dogs (Group 7, Section 1)	2.74	2.11–3.57
	British and Irish Pointers and Setters (Group 7, Section 2)	2.74	2.10–3.59
	Retrievers (Group 8, Section 1)	2.45	1.96–3.07
	Flushing dogs (Group 8, Section 2)	1.80	1.41–2.30
	Water dogs (Group 8, Section 3)	1.90	1.40–2.57
	Bichons and related breeds (Group 9, Section 1)	0.73	0.56–0.95
	Poodle (Group 9, Section 2)	0.91	0.71–1.18
	Small Belgian dogs (Group 9, Section 3)	10.04	0.52–64.1
	Hairless dogs (Group 9, Section 4)	1.68	0.87–3.05
	Tibetan breeds (Group 9, Section 5)	0.91	0.70–1.20
	Chihuahueno (Group 9, Section 6)	0.42	0.31–0.57
	English toy Spaniels (Group 9, Section 7)	0.79	0.56–1.10
	Japan Chin and Pekingese (Group 9, Section 8)	0.73	0.47–1.13
	Continental toy Spaniel and others (Group 9, Section 9)	1.07	0.74–1.53
	Kromfohrländer (Group 9, Section 10)	2.48	1.18–4.81
	Small molossian type dogs (Group 9, Section 11)	2.38	1.86–3.05
	Long-haired or fringed Sighthounds (Group 10, Section 1)	3.45	2.38–4.98
	Rough-haired Sighthounds (Group 10, Section 2)	6.88	4.03–11.5
	Short-haired Sighthounds (Group 10, Section 3)	1.63	1.20–2.20

IRR: incidence rate ratio; 95%CI: 95% confidence interval.

## Discussion

This study of canine tumor IRs is based on 54’986 tumors with information on age, sex, breed, and topographical body location collected over a period of 13 years in four Swiss veterinary pathology laboratories. Diagnoses were coded according to the novel Vet-ICD-O-canine-1 guidelines [29], and mandatory dog registration data from the Swiss animal registration database constituted the denominator population. To our knowledge, this represents the largest study to date on breed-specific canine tumor IRs. It complements and extends the results of a previous study based on the SCCR by determining the IRs for benign and malignant tumors in all topographical locations and over a longer period of time [4]. In addition, a negative binomial regression model was run to provide IRRs for malignant tumors (Table 6).

The IR determined in our study was 775/100’000 DYAR for all tumors and 338/100’000 DYAR for malignant neoplasms. The dataset comprised samples of dogs from all 26 Swiss cantons submitted by primary care veterinarians, referral centers and veterinary teaching hospitals. However, the tumor incidence in the Swiss canine population was underestimated, mainly because data from other diagnostic laboratories operating in Switzerland were not available and not all tumors commonly undergo pathological examination. The range of IRs varies between studies, with previous publications reporting similar values for tumors [4, 23] and cancers [19] or markedly lower IRs [9, 10]. The inclusion of necropsy and cytological samples likely allowed for a better representation of tumors affecting deep tissues and organs in our analysis. In contrast, an Italian survey based on the Piedmont Canine Cancer Registry (2001–2008), a U.K. study on tumor insurance claims (1997–1998), and a hospital-based U.S. study (1972–1973) reported substantially higher IRs (1’701/100’000 DYAR [11]; 1’948/100’000 dogs per year [16] and 1’416/100’000 dogs [24], respectively). Especially in the latter two studies, a thorough investigation can be assumed, leading to an accurate assessment of the tumor burden in the indicated reference population. Notably, Cushing’s syndrome was considered a neoplasm in the U.K. study, which may further explain higher IRs and underscores the importance of uniform inclusion criteria. In general, many further factors can hamper the comparability of IRs between studies. They include differences in age structure, breed distribution, and neutering status as well as non-animal related factors such as sociodemographic and economic aspects related to the owner’s financial situation and the dog’s status as a family member, and geographical aspects, which may have an impact on the dog’s access to veterinary care [44]. Additionally, environmental factors such as exposure to certain substances, e.g., toxic chemicals, likely influence tumor IRs [45, 46]. Finally, previous studies calculated IRs per DYAR [4, 9, 11, 16] or per dogs [10, 19, 24]. The latter method often uses denominators that are based on the mid-year population and are considered approximately equivalent [38]. Although these issues limit the comparability of absolute values of IRs across studies, IRs are very useful for within-study comparisons.

The percentage of benign tumors was higher than that of malignant tumors (54.91% versus 45.09%, respectively), as reported in most previous studies in dogs [4–6, 9, 11, 16, 18, 23], but different from a Japanese [21] and two Italian [8, 10] studies, which reported 57.5%, 53%, and 51% of all tumors as malignant, respectively. The percentage of benign tumors may generally be underestimated due to less frequent pathological assessment. Many factors can influence malignancy proportions, limiting comparisons between studies. More substantial differences in the distribution of benign and malignant tumors have been observed between species, specifically between dogs and cats [6, 10]. However, we show distinct variations in the proportions and IRs of benign and malignant tumors between dog breeds.

As reported in some registry-based studies [11, 19], the overall tumor IR and the cancer incidence rate ratios (IRRs) were higher in several purebred breeds compared to mixed breeds (Tables 3 and 6). The latter had a 0.75-fold lower overall tumor IR than the average IR for all breeds. Inbreeding patterns frequently occur in purebred dogs [47, 48], and inbreeding has been reported to negatively affect the life expectancy of dogs [49], which might be attributable to higher susceptibility to hereditary diseases, including cancer. Other studies found no differences in the OR [22] or prevalence [20] of tumors between mixed breed and purebred dogs. In contrast, the previous SCCR cutaneous study found a higher IR in mixed breeds (979) compared to the average IR for all dogs (372) [4]. We could not confirm this result in our analysis which revealed an IR for cutaneous tumors of 289 and 331 in mixed breeds vs. 414 and 424 as average of all breeds for the periods 2008–2013 and 2008–2020, respectively.

In contrast to other studies that focused on popular dog breeds [4, 11, 19, 21], our analysis included breeds with relatively few individuals (≥200 dogs registered in Switzerland for the calculation of IRs and all breeds for the calculation of IRRs) in the reference population. This approach revealed meaningful and partly novel differences of IRs between dog breeds (Tables 3 and 4) and IRRs between breed groups and sections (Table 6). The highest overall tumor IR (>4 times higher than the average for all breeds) was found in the PON, which is a novel finding in a relatively uncommon breed. It was associated with increased IRs for benign basal cell neoplasms, lipomas, adnexal adenomas, but also for adenocarcinomas, and malignant lymphomas. Of note, the 95%CIs for these IRs are relatively wide. Thus, the results should be interpreted with caution and confirmed by further studies. For the breeds with the following top four IRs, already known predispositions were confirmed. They included relatively high IRs for lipomas [50], melanocytomas [14], and melanomas [4] in Magyar Vizslas; a markedly increased IR for histiocytic sarcomas (IR: 336, 48.6-fold higher than the average for all breeds) [4, 17] and for canine cutaneous histiocytomas (CCH) [51] in FCRs; high IRs for squamous cell carcinomas (IR: 459, 33.9-fold higher than average) [52], melanocytomas, and melanomas in Giant Schnauzers [14]; and high risk for the development of lipomas [53] and CCHs [14] in Doberman Pinschers.

Negative binomial regression analysis provides the ability to compare IRs for FCI breed groups and sections with mixed breeds, adjusting for age and sex. The highest IRRs for malignant tumors were found for Nordic hunting dogs, rough-haired Sighthounds, and Cattledogs (8.08, 95%CI 3.55–16.7; 6.88, 95%CI 4.03–11.5; and 3.81, 95%CI 2.43–5.88, respectively). For the IRR of Nordic hunting dogs, a bias resulting in a wide 95%CI is likely because there were only nine malignant tumors in this group and section (S7 Table), all of which were diagnosed in breeds with less than 200 individuals registered in Switzerland. Among rough-haired Sighthounds, both Irish Wolfhounds and Deerhounds had above-average IRs for malignant tumors (IRs: 635 and 376, respectively), and bone tumors (Table 5), as described in other studies [54–56]. Among Cattledogs, the Bouvier des Flandres had high IRs for malignant tumors (IR: 1’171, Table 4, S9 Table) and for hemangiosarcomas (IR: 127, Table 5), whereas the Australian Cattle Dog had a below average cancer IR of 231. No malignant tumors were diagnosed in the remaining breeds in this breed group and section. This suggests an etiologic heterogeneity within groups and sections in the development of malignant tumors that requires investigation at the individual breed level. Interestingly, the three breed groups and sections with the lowest IRRs for malignant tumors compared to mixed breeds include small dog breeds, which is consistent with a recent study [20] and with the finding that small dogs are less likely to die from tumors [2]. In general, it should also be noted that the composition of the mixed breed group used as a reference is unknown (e.g., Swiss Mountain- and Cattledogs are comparatively popular in Switzerland), which limits the interpretation. Another possible source of bias in the regression model presented, the magnitude of which we cannot currently estimate, is that the DYAR refer to the entire dog population and do not take into account disease onset as an endpoint of risk exposure.

Cancer was described as the leading cause of death in a U.K. study, especially in the FCR (54.3%), the Bernese Mountain Dog (BMD) (45.7%), and the Giant Schnauzer (41.0%) [1], which is in line with our results of high overall and malignant tumor IRs in these breeds (Tables 3 and 4, S9 Table). Other studies found the highest cancer rates [21], a high SMR for malignant tumors [5], as well as the highest tumor mortality rate [57] in the BMD. Furthermore, a Swiss study examining causes of death in the BMD concluded that histiocytic sarcomas and lymphomas were the tumors primarily involved [58]. Histiocytic sarcoma accounts for approximately 1/7 of all deaths in BMDs and FCRs [59]. The high susceptibility to histiocytic sarcoma is most likely attributable to a genetic background. For example, several chromosomal risk loci were reported to correlate with development of histiocytic sarcoma in these breeds [60]. Epidemiological studies establishing risk at the breed level can potentially inform such genetic studies. In this context, it should be emphasized that the identification of breeds with low tumor incidence in particular depends on a sufficient number of individuals in the reference population. While the absence of tumor samples may indicate a lower risk of tumor development, IRs may also be underestimated. Of note, only seven of the 112 breeds registered in Amicus and not represented in the tumor dataset (S3 Table) comprised ≥200 dogs registered in Switzerland. In addition, breed misclassification should be noted as a potential source of error that occurs at various stages of data collection and processing.

More than 50% of the tumors in our dataset derived from dogs between seven and eleven years of age, consistent with the SCCR cutaneous study, where over 50% of all tumors were found in eight to eleven-year-old dogs [4]. IRs of malignant tumors peaked at a higher age (eleven years) than benign tumors (ten years; Fig 2), in agreement with other studies [5, 6, 8, 61]. Similar to our results, a Portuguese study found the highest number of tumors in ten-year-old dogs [7]. Recently, it has been hypothesized that the increasing risk of tumor development with age described for dogs and humans might derive from the inability of tumor protection mechanisms to adapt to a rapidly increasing life expectancy [62]. Consistent with our results, a decrease in IRs has been demonstrated in very old humans and dogs [62]. Omission of diagnostic pathology while electing euthanasia might contribute to this effect in old dogs.

In accordance with previous studies, female dogs showed higher overall tumor numbers [5, 6, 8, 17, 18, 20, 22] and IRs [4, 9–11] compared to male dogs (IR: 850 vs. 679, respectively), and a higher IRR for malignant tumors (Table 6). These results may be partly influenced by relatively high numbers of tumors in the mammary gland. In our study, 7’794 of 7’974 mammary gland tumors were found in females (97.74%), of which 5’596 (70.18%) were diagnosed in intact females. In other studies, intact females similarly exhibited increased risk for mammary gland tumors, compared to neutered females [15, 19]. This organ was frequently found among the three most common tumor locations in various studies [5, 7, 8, 10, 16–19, 23, 26] and ranked third in our study, accounting for 14.50% of all topographical sites. In contrast, the proportion of mammary gland tumors was markedly lower (2%) in a recent U.S. study [20], which is commonly attributed to the fact that most dogs in the United States are neutered [20, 63]. While our data are not suited to further pinpoint the influence of neutering on this tumor type, it is of note that differences in the distribution of sex (and neutering status) were also apparent in extra-mammary tumor groups. For example, mast cell tumors (MCT) in female neutered dogs accounted for over one third of all MCT cases in this study, complementing the results of a study that found markedly increased ORs for MCTs in female neutered dogs [64]. Our findings that CCHs occurred more frequently in male (IRs: 53) than in female dogs (IRs: 37) were supported by other studies [7, 17]. The reasons for the differences in tumor risk between sexes are not well understood.

Most tumors were found in the skin (n = 19’045; 34.64%), which is consistent with the results from previous studies in which the skin was the most frequently affected site [5–8, 15, 20, 23]. The fact that tumors in the skin are easier to detect and sample than tumors in deeper tissues may contribute to this finding. Soft tissue was the second most common topographical site (20.17%) in the present study, which is in line with a study from the United States (16.02%) [20]. However, several studies did not distinguish between skin and soft tissue [4, 10, 11, 16, 17, 21, 26], limiting comparability.

Out of the ten most common tumor groups in this study (Table 2), *lipomas [out of 885–888]*, *CCHs [9751*.*1/0]*, and *complex mixed and stromal adenomas [out of 893–899]* are benign. Lipomas (IR: 110; 14.27% of all tumors) were common in previous reports, accounting for 5.8 to 12.5% of all tumors [4, 5, 7, 15, 16]. Their high percentage in our study may be due to the inclusion of cytological diagnoses. CCHs are frequent neoplasms in young dogs [4, 16, 17, 65]. In our study, the highest IRs for CCHs were found in FCRs, Boxers, and Doberman Pinschers, as previously reported [4, 14, 66], but also in Russian Black Terriers and Dogo Canarios. Among the complex mixed and stromal adenomas, *benign mixed tumors [8940/0]*, *complex adenomas [8983/0]*, and *myoepitheliomas [8982/0]* were most frequent (S6 Table). They were often diagnosed in the Field Spaniel, the Russian Black Terrier, and the Welsh Springer Spaniel. The majority of these tumors were associated with the *mammary gland [C50]* (n = 2’940; 99.19%) and occurred largely in females (n = 2’907; 98.08%).

Most of the remaining tumors in the group of the ten most common tumor groups in this study are malignant or show a propensity for malignancy. *MCTs [974]* (IR: 77; 9.98% of all tumors) ranked high in frequency [4, 5, 7, 15] or IRs (60–126) [4, 16] in several studies. We found the highest IRs for MCTs in Dogo Argentinos, Boxers, Nova Scotia Duck Tolling Retrievers, and Rhodesian Ridgebacks. The latter three breeds, particularly Boxers and Rhodesian Ridgebacks, have been found to be at high or increased risk for MCTs in several other studies [4, 22, 23, 28, 64, 67, 68]. To the author’s knowledge, the Dogo Argentino has not yet been associated with an increased risk for the development of MCTs.

*Adenocarcinomas [814–838]* showed the highest IRs in the Field Spaniel, the PON, and the Gordon Setter. Over two-thirds of all adenocarcinomas in this study (1’871; 70.10%) were found in female intact and neutered dogs and most of them originated from the *mammary gland [C50]* (n = 1’260; 47.21%). The Field Spaniel presented the highest IRs for both complex mixed and stromal adenomas and adenocarcinomas. This breed has not yet been reported to be at higher risk. However, in a Swedish study on mammary tumors, the English Springer Spaniel was identified as the breed with the highest IR [69] and in a Mexican study the Cocker Spaniel was the second most affected breed [70]. Our data support a heritable component in mammary tumorigenesis shared by the breeds belonging to the same breed group (8: Retrievers—Flushing Dogs—Water Dogs) and section (2: Flushing Dogs), according to the FCI [42].

The IR for *lymphomas [959–972]* in the present study was 36, which is similar to the findings of other studies (range: 19.9-33/100’000 DYAR or dogs) [9, 19, 71]. King Charles Spaniels, Manchester Terriers, PONs, Dogues de Bordeaux, and Airedale Terriers had the highest IRs for this tumor group. Increased ORs have been reported for the latter two breeds [72, 73]. Interestingly, the King Charles Spaniel was found to have a reduced risk of lymphoid neoplasms in an Australian study [73]. This discrepancy might be explained by differences in the genetic background of different populations, which justifies the assessment of tumor IRs at a local scale.

*Melanomas [*out of *872–879]* were frequently diagnosed in the Giant Schnauzer, Magyar Vizsla, and Airedale Terrier, breeds that have been described as more likely to develop melanoma than others [4, 28]. However, this was not the case for the Russian Black Terrier (IR: 345). *Melanocytomas [out of 872–879]* exhibited the highest IRs in Magyar Vizslas, Nova Scotia Duck Tolling Retrievers, Australian Terriers, Irish Terriers, and Rhodesian Ridgebacks, which is in line with previous studies [4, 14, 66].

Other relatively commonly diagnosed malignant tumors included *bone tumors*, *blood vessel tumors*, *gliomas*, *and transitional cell carcinomas*. *Osseous and chondromatous neoplasms [918–924]* showed an IR of 8, while a Swedish study based on insurance data found an IR of 5.5/10’000 DYAR [54], whereas a U.S. study found an IR of 7.9/100’000 DYAR [19], which is consistent with our results. We found a ten to 20-fold higher IR than the average in Deerhounds (IR: 150; 95%CI 18–541), Rottweilers (IR: 93; 95%CI 62–135), Borzois (IR: 88; 95%CI 24–225), Irish Wolfhounds (IR: 86; 95%CI 18–251), and Landseers (IR: 74; 95%CI 15–216). All of these breeds, with the exception of the Landseer, have been described as having a higher risk of developing osteosarcoma [17, 22, 54–56, 74]. Bouvier des Flandres showed the highest IR (127; 95%CI 26–371) for *hemangiosarcomas*, *NOS [9120/3]*, followed by the Dogo Argentino (IR: 117; 95%CI 38–274), the Leonberger (IR: 106; 95%CI 58–177), the Dogue de Bordeaux (IR: 105; 95%CI 34–246), and the Cane Corso Italiano (IR: 101; 95%CI 46–193). The Dogo Argentino also showed an increased IR for benign blood vessel tumors (IR: 140; 95%CI 52–306). Most *gliomas [938–948]* in the dataset were classified as malignant. Bearded Collies, Cane Corso Italianos, Boxers, French Bulldogs, and Bullmastiffs had the highest IRs for this tumor group. Only the latter three breeds are known to be predisposed to develop glial neoplasms [13, 75, 76]. *Transitional cell papillomas and carcinomas [812–813]* mainly consisted of *urothelial carcinomas*, *NOS [8120/3]* (98.15%) found in the *bladder*, *NOS [C67*.*9]* and *urethra [C68*.*0]* of female dogs (n = 181; 67.04%), with over two fifth of the tumors found in neutered females (n = 110; 40.74%). A ratio of 1.7(female):1(male) affected by urothelial carcinoma has been described [77], which is in line with a ratio of 1.67(f):1(m) in our study. In accordance with the results of a previous study [77], Scottish Terriers had the highest IR for this tumor group, with a 44-fold higher IR for urothelial carcinoma compared to mixed breed dogs.

In summary, our study based on a large diagnostic pathology-derived dataset essentially confirms published data and adds to the knowledge of canine tumor IRs, including those for benign and malignant tumors, and reveals novel breed predispositions. Differences in data collection, coding, structuring, and analysis as well as other confounding factors such as inclusion criteria, population structure, and economic, geographic, and environmental factors hinder comparisons across studies. Furthermore, denominator data, obtained through owner surveys [10, 11, 19, 21], national kennel clubs [17], insurance companies [16], or veterinary hospitals [24] can introduce confounders, including response and sampling bias, exclusion of mixed breed dogs, skewed age and breed distribution, and health bias, respectively. In our study, denominator data representing the entire population allowed for optimal exploitation of diagnostic data, enabling the calculation of IRs and IRRs while accounting for age, sex, and breed. In the future, precise linkage to the denominator population and inclusion of more detailed patient information, along with data from additional pathology laboratories in Switzerland, would greatly improve tumor risk assessments based on SCCR data. Such epidemiological data may be of importance not only for the diagnostic work-up of patients and for breeding management, but also for veterinary and comparative oncology research, providing insights into tumorigenesis and potentially yielding new directions for cancer prevention strategies including early screening.

## Supporting information

S1 Table Keywords applied on the original diagnosis field to identify tumor diagnoses. (CSV)

S2 Table Behavioral classification (benign or malignant) of tumors initially coded *uncertain whether benign or malignant [/1]* or *in situ [/2]*. ^A^Uncertain diagnosis regarding behavioral and prognostic classification of tumor; ^B^Small numbers of tumor type; ^C^Described as (frequently/usually) benign; ^D^Rare (<10%) occurrence of metastasis observed; ^E^*In situ* [/2] was assigned to neoplasms indicating their potential of malignant progression, exception: bronchioloalveolar adenoma (synonym: adenocarcinoma in situ in the lung) is described as benign; ^F^In situ [/2] was assigned to neoplasms indicating their potential of malignant progression; ^G^Described as malignant; ^H^Frequent (>10%) occurrence of metastasis observed; ^I^Frequent recurrences observed; *Preferred term indicated in the Vet-ICD-O-canine-1 was changed to a term more closely matching the tumor entity described in the diagnoses, **Code and/or term not available in Vet-ICD-O-canine-1 and assigned based on pathological tumor diagnosis; NOS: not otherwise specified; 1. Meuten DJ. Tumors in domestic animals. 5th ed. Donald J. Meuten, editor. Raleigh, NC, USA: John Wiley & Sons, Inc.; 2017; 2. Meningioma Grading. [cited 12 May 2023]. Available: https://www.hopkinsmedicine.org/health/conditions-and-diseases/meningioma-grading; 3. Stephen J. Withrow, Rodney Page, David M. Vail. Withrow & MacEwen’s small animal clinical oncology. 5th ed. David M. Vail, editor. Elsevier; 2013; 4. Bellamy E, Berlato D. Canine cutaneous and subcutaneous mast cell tumours: a narrative review. J Small Anim Pract. 2022;63: 497–511. doi:10.1111/jsap.13444.
(PDF)

S3 Table Dog breeds registered in Amicus not diagnosed (X) with any of the ten most common tumor groups and/or not represented at all in the Swiss Canine Cancer Registry (2008–2020). N: number; NOS: not otherwise specified.
(PDF)

S4 Table The ten most common tumor groups in the Swiss Canine Cancer Registry (2008–2020) with absolute frequency in each topographical location. N: number.
(PDF)

S5 Table Absolute distribution of each morphologic tumor category per topographical site where *other tumors* accounted for more than 60% of all tumors, compared to the ten most common tumor types. The tumor categories that formed the largest group per each topographical site are emphasized in **bold**.
(PDF)

S6 Table Morphological tumor diagnoses, absolute frequency, and incidence rates per 100’000 dog-years at risk of the 54’986 tumors registered in the Swiss Canine Cancer Registry, 2008–2020. IR: incidence rate (tumors per 100‘000 dog-years at risk); N: number; 95%CI: 95% confidence interval; [/0]: benign, [/1]: uncertain whether benign or malignant, [/2]: in situ, [/3]: malignant; B: biopsy, C: cytology, Ne: necropsy; NOS: not otherwise specified; *Preferred term indicated in the Vet-ICD-O-canine-1 was changed to a term more closely matching the tumor entity described in the diagnoses; **Code and/or term not available in Vet-ICD-O-canine-1 and assigned based on pathological tumor diagnosis.
(PDF)

S7 Table Results from the negative binomial regression model showing the incidence rate ratios of 21’803 malignant tumors in the Swiss Canine Cancer Registry (2008–2020), absolute tumor numbers, and dog-years at risk for each value and measures of goodness of fit. IRR: incidence rate ratio; 95%CI: 95% confidence interval; Std. Error: standard error; N: number; (na.omit): number of tumors/DYAR after excluding cases/dogs with missing information on one or more variables; DYAR: dog-years at risk; AIC: Akaike information criterion.
(PDF)

S8 Table The 20 Swiss dog breeds (precise breed) with the highest incidence rates for benign tumors between 2008 and 2020 and their respective Swiss Canine Cancer Registry data. IR: incidence rate (tumors per 100‘000 dog-years at risk); N: number; 95%CI: 95% confidence interval; DYAR: dog-years at risk.
(PDF)

S9 Table The 20 Swiss dog breeds (precise breed) with the highest incidence rates for malignant tumors between 2008 and 2020 and their respective Swiss Canine Cancer Registry data. IR: incidence rate (tumors per 100‘000 dog-years at risk); N: number; 95%CI: 95% confidence interval; DYAR: dog-years at risk.
(PDF)
