# Supplementary material for: Incidence rates of the most common canine tumors based on data from the Swiss Canine Cancer Registry (2008 to 2020)
Source: PLoS One. 2024 Apr 18;19(4):e0302231. doi: 10.1371/journal.pone.0302231 (PMC11025767; doi:10.1371/journal.pone.0302231)
Supplement: S2 Table — AUncertain diagnosis regarding behavioral and prognostic classification of tumor; BSmall numbers of tumor type; CDescribed as (frequently/usually) benign; DRare (<10%) occurrence of metastasis observed; EIn situ [/2] was assigned to neoplasms indicating their potential of malignant progression, exception: bronchioloalveolar adenoma (synonym: adenocarcinoma in situ in the lung) is described as benign; FIn situ [/2] was assigned to neoplasms indicating their potential of malignant progression; GDescribed as malignant; HFrequent (>10%) occurrence of metastasis observed; IFrequent recurrences observed; *Preferred term indicated in the Vet-ICD-O-canine-1 was changed to a term more closely matching the tumor entity described in the diagnoses, **Code and/or term not available in Vet-ICD-O-canine-1 and assigned based on pathological tumor diagnosis; NOS: not otherwise specified; 1. Meuten DJ. Tumors in domestic animals. 5th ed. Donald J. Meuten, editor. Raleigh, NC, USA: John Wiley & Sons, Inc.; 2017; 2. Meningioma Grading. [cited 12 May 2023]. Available: https://www.hopkinsmedicine.org/health/conditions-and-diseases/meningioma-grading; 3. Stephen J. Withrow, Rodney Page, David M. Vail. Withrow & MacEwen’s small animal clinical oncology. 5th ed. David M. Vail, editor. Elsevier; 2013; 4. Bellamy E, Berlato D. Canine cutaneous and subcutaneous mast cell tumours: a narrative review. J Small Anim Pract. 2022;63: 497–511. doi:10.1111/jsap.13444. (PDF) [file pone.0302231.s002.pdf]

S2 Table. Behavioral classification (benign or malignant) of tumors initially coded uncertain whether benign or malignant [/1] or in situ [/2].

Tumor Code and Type	Classification
8000/1 Neoplasm, uncertain whether benign or malignant	excluded[A]
8004/1 Tumor, spindle cell type, NOS**	excluded[A]
8006.1/1 Round cell tumor, NOS	excluded[A]
8010/1 Epithelial tumor, NOS**	excluded[A]
8010/2 Carcinoma in situ, NOS	malignant[F][1]
8070/2 Squamous cell carcinoma in situ	malignant[F][1]
8120/2 Urothelial carcinoma, in situ	malignant[F][1]
8140/2 Adenocarcinoma in situ*	malignant[E][1]
8170/1 Hepatocellular neoplasm, NOS**	excluded[A]
8246/1 Neuroendocrine neoplasm, NOS**	excluded[A]
8345/1 C cell neoplasm, NOS**	excluded[A]
8401.2/1 Anal sac neoplasm, NOS**	excluded[A]
8401/1 Apocrine neoplasm, NOS**	excluded[A]
8410.1/1 Meibomian gland epithelioma	benign[D][1]
8410.2/1 Hepatoid gland epithelioma	benign[D][1]
8410.2/1 Hepatoid gland neoplasm, NOS*	excluded[A]
8410/1 Sebaceous epithelioma	benign[D][1]
8420/1 Ceruminous neoplasm, NOS**	excluded[A]
8490/2 Signet-ring cell carcinoma in situ**	malignant[F][1]
8500/2 Ductal carcinoma in situ**	malignant[F][1]
8503/2 Intraductal papillary carcinoma in situ**	malignant[F][1]
8590/1 Sex cord-gonadal stromal tumor, NOS	excluded[A]
8640/1 Sertoli cell tumor, NOS	benign[C][1]
8650/1 Interstitial cell tumor, NOS	benign[C][1]
8691/1 Aortic body tumor, NOS	benign[C][1]
8720/1 Melanocytic neoplasm, NOS**	excluded[A]
8800.0/1 Soft tissue tumor, NOS	excluded[A]
8815.1/1 Canine perivascular wall tumor, NOS	malignant[I][1]
8940/1 Mixed tumor, NOS**	excluded[A]
8983.1/2 Complex carcinoma in situ**	malignant[F][1]
8983/1 Complex neoplasm, NOS**	excluded[A]
9061/1 Seminoma, NOS	benign[C/D][1]
9073/1 Gonadoblastoma	excluded[B]
9080/1 Teratoma, NOS	benign[C][1]
9270/1 Odontogenic tumor, NOS**	benign[D][1]
9310.2/1 Acanthomatous ameloblastoma	excluded[B]
9390/1 Choroid plexus tumor	excluded[A]
9391/1 Cellular ependymoma	excluded[B]
9421/1 Pilocytic astrocytoma	excluded[B]
9490/1 Ganglioneuroma	excluded[B]
9505/1 Ganglioglioma, NOS	excluded[B]
9530.0/1 Meningioma, NOS	excluded[A]
9530.1/1 Meningioma, NOS, WHO grade I	benign[C][1]
9530.2/1 Microcystic meningioma	benign[C][1]
9531/1 Meningothelial meningioma	benign[C][1]
9532/1 Fibrous meningioma	benign[C][1]
9533/1 Psammomatous meningioma	benign[C][1]
9537/1 Transitional meningioma	benign[C][1]
9539/1 Meningioma, WHO grade II	malignant[G][2]
9563/1 Nerve sheath tumor, NOS	malignant[G][3]
9734/1 Plasmacytoma, extramedullary (digestive tract)	benign[C][1]
9734/1 Plasmacytoma, NOS**	benign[C][1]
9740.0/1 Subcutaneous mast cell tumor	benign[D][4]
9740.1/1 Cutaneous mast cell tumor grade Patnaik I	benign[D][4]
9740.2/1 Cutaneous mast cell tumor grade Patnaik II	malignant[H][4]
9740.3/1 Cutaneous mast cell tumor grade Patnaik III	malignant[H][4]
9740.4/1 Cutaneous mast cell tumor grade Kiupel low	malignant[H][4]
9740.5/1 Cutaneous mast cell tumor grade Kiupel high	malignant[H][4]
9740.6/1 Cutaneous mast cell tumor grade Patnaik I, Kiupel low	benign[D][4]
9740.7/1 Cutaneous mast cell tumor grade Patnaik II, Kiupel low	malignant[H][4]
9740.8/1 Cutaneous mast cell tumor grade Patnaik II, Kiupel high	malignant[H][4]
9740.9/1 Cutaneous mast cell tumor grade Patnaik III, Kiupel high	malignant[H][4]
9740/1 Mast cell tumor, NOS	malignant[H][4]
9766/1 Lymphomatoid granulomatosis	excluded[B]

A: Uncertain diagnosis regarding behavioral and prognostic classification of tumor; B: Small numbers of tumor type; C: Described as (frequently/usually) benign; D: Rare (<10%) occurrence of metastasis observed; E: In situ [/2] was assigned to neoplasms indicating their potential of malignant progression, exception: bronchioloalveolar adenoma (synonym: adenocarcinoma in situ in the lung) is described as benign; F: In situ [/2] was assigned to neoplasms indicating their potential of malignant progression; G: Described as malignant; H: Frequent (>10%) occurrence of metastasis observed; I: Frequent recurrences observed; *Preferred term indicated in the Vet-ICD-O-canine-1 was changed to a term more closely matching the tumor entity described in the diagnoses, **Code and/or term not available in Vet-ICD-O-canine-1 and assigned based on pathological tumor diagnosis; NOS: not otherwise specified; 1. Meuten DJ. Tumors in domestic animals. 5th ed. Donald J. Meuten, editor. Raleigh, NC, USA: John Wiley & Sons, Inc.; 2017; 2. Meningioma Grading. [cited 12 May 2023]. Available: https://www.hopkinsmedicine.org/health/conditions-and-diseases/meningioma-grading; 3. Stephen J. Withrow, Rodney Page, David M. Vail. Withrow & MacEwen's small animal clinical oncology. 5th ed. David M. Vail, editor. Elsevier; 2013; 4. Bellamy E, Berlato D. Canine cutaneous and subcutaneous mast cell tumours: a narrative review. J Small Anim Pract. 2022;63: 497–511. doi:10.1111/jsap.13444.
