# Supplementary material for: Incidence rates of the most common canine tumors based on data from the Swiss Canine Cancer Registry (2008 to 2020)
Source: PLoS One. 2024 Apr 18;19(4):e0302231. doi: 10.1371/journal.pone.0302231 (PMC11025767; doi:10.1371/journal.pone.0302231)
Supplement: S3 Table — N: number; NOS: not otherwise specified. (PDF) [file pone.0302231.s003.pdf]

S3 Table. Dog breeds registered in Amicus not diagnosed (X) with any of the ten most common tumor groups and/or not represented at all in the Swiss Canine Cancer Registry (2008-2020).

Dog breed	N dogs (Amicus)	Breed not represented in	
		10 most common	All tumors
Aïdi	23	X	X
American Foxhound	6	X	X
American Hairless Terrier	41	X	X
American Water Spaniel	6	X	X
Anglo-Français de petite vénerie	19	X	
Ariégeois	23	X	X
Australian Stumpy Tail Cattle Dog	9	X	X
Basset artésien normand	60	X	
Basset bleu de Gascogne	22	X	X
Basset Griffon Vendéen NOS	128	X	
Berger de Savoie	30	X	
Bouvier des Ardennes	14	X	X
Braque d'Auvergne	70	X	
Braque de l'Ariège	2	X	X
Braque du Bourbonnais	67	X	X
Braque français	42	X	X
Braque Saint-Germain	17	X	X
Briquet Griffon Vendéen	57	X	X
Ca de Bestiar	33	X	X
Canadian Eskimo Dog	44	X	X
Cão de gado transmontano	7	X	X
Cão Fila de São Miguel	28	X	X
Carolina Dog	4	X	X
Český fousek	28	X	X
Český Teriér	88	X	X
Chien d'Artois	1	X	
Chinook	4	X	X
Cimarrón Uruguayo	6	X	X
Ciobănesc Românesc Carpatin	24	X	X
Ciobănesc Românesc de Bucovina	13	X	X
Ciobănesc Românesc Mioritic	15	X	X
Clumber Spaniel	44	X	
Coonhound NOS	6	X	
Crnogorski Planinski Gonič	2	X	X
Cursinu	24	X	X
Dansk-Svensk Gårdshund	61	X	X
German Roughhaired Pointer	22	X	X
Dingo	3	X	X
Srpski trobojni gonič	2	X	X
Drever	3	X	X
Dunker	53	X	X
Ellenikós Poimenikós	27	X	X
English Foxhound	9	X	X
Epagneul de Pont-Audemer	2	X	X
Suomenajokoira	4	X	X
Suomenpystykorva	32	X	X
Français blanc et noir	3	X	X
Français blanc et orange	5	X	X
Braque français	34	X	X
Galgo Español	2'613	X	X
Gammel Dansk Honsehund	5	X	X
Gascon Saintongeois	5	X	X
Germanischer Bärenhund	16	X	X
Gończy Polski	22	X	X
Grand anglo-français blanc et orange	2	X	X
Grand anglo-français blanc et noir	1	X	X
Grand anglo-français tricolore	2	X	X
Grand Gascon Saintongeois	4	X	X
Greyster	53	X	X
Griffon Bruxellois	116	X	X
Griffon Fauve de Bretagne	108	X	X
Griffon Nivernais	58	X	X
Hahoawu	4	X	
Haldenstøver	1	X	X
Hamiltonstövare	6	X	X
Harrier	3	X	X
Hellinikos Ichnilatis	55	X	X

Dog breed	N dogs (Amicus)	Breed not represented in	
		10 most common	All tumors
Hokkaido	3	X	X
Hollandse Smoushond	3	X	X
Hygenhund	1	X	X
Istarski kratkodlaki gonič	18	X	
Istarski oštrodlaki gonič	6	X	X
Swedish Elkhound	6	X	X
Japan Chin	213	X	
Nihon Teria	4	X	X
Kai	4	X	X
Kintamani-Bali-Dog	5	X	X
Kishu	9	X	X
Komondor	42	X	
Korea Jindo Dog	13	X	
Kraški ovčar	5	X	X
Kritikos Lagonikos	14	X	X
Hrvatski ovčar	29	X	
Lancashire Heeler	4	X	X
Lapinporokoira	86	X	X
Louisiana Catahoula Leopard Dog	20	X	X
Lurcher	26	X	X
Majorero Canario	35	X	
Mastín del Pirineo	24	X	
Bankhar Dog	11	X	X
New Zealand Huntaway	5	X	X
New Zealand Heading Dog	14	X	X
Norrbottenspets	8	X	X
Norsk Buhund	25	X	
North American Shepherd	10	X	X
Northern Inuit	30	X	X
Austrian Pinscher	35	X	X
Pachon Navarro	9	X	X
Pye-Dog	2	X	X
Perdiguero de Burgos	2	X	X
Petit Gascon Saintongeois	9	X	X
Podenco Andaluz	84	X	
Podengo Português	505	X	
Poitevin	11	X	X
Porcelaine	34	X	X
Posavski Gonič	16	X	X
Pudelpointer	49	X	X
Rafeiro do Alentejo	19	X	X
Ratier	556	X	X
Ratier du Jura	562	X	X
Russo-European Laika	18	X	X
Sabueso Español	21	X	X
Schillerstövare	4	X	
Swedish Lapphund	7	X	X
Segugio Maremmano	16	X	X
Shikoku	37	X	X
Shiloh Shepherd Dog	20	X	X
Skye Terrier	61	X	
Slovenský čuvač	59	X	X
Slovenský hrubosrstý stavač	17	X	X
Slovenský kopov	212	X	X
Bosanski Oštrodlaki Gonič – Barak	17	X	X
Taiwan Dog	5	X	X
Thai Bangkaew Dog	3	X	X
Tornjak	31	X	X
Tosa	19	X	X
Treibhund	9	X	X
East Siberian Laïka	6	X	X
Welsh Corgi Pembroke	544	X	
West Siberian Laika	62	X	X
Westphalian Dachsbracke	30	X	X
Wetterhoun	5	X	X
Yakutian Laika	16	X	X
Central Asian Shepherd Dog	42	X	X

N: number; NOS: not otherwise specified.
