# Supplementary material for: Incidence rates of the most common canine tumors based on data from the Swiss Canine Cancer Registry (2008 to 2020)
Source: PLoS One. 2024 Apr 18;19(4):e0302231. doi: 10.1371/journal.pone.0302231 (PMC11025767; doi:10.1371/journal.pone.0302231)
Supplement: S4 Table — N: number. (PDF) [file pone.0302231.s004.pdf]

S4 Table. The ten most common tumor groups in the Swiss Canine Cancer Registry (2008–2020) with absolute frequency in each topographical location.

Topography [code]	Lipomas*	Mast cell tumors*	Canine cutaneous histiocytomas*	Complex mixed and stromal adenomas*	Adnexal and skin appendage adenomas*	Adenocarcinomas*	Lymphomas*	Basal cell neoplasms, benign*	Melanomas*	Sarcomas*	Sum of (*)	N all tumors	Other tumors	
	[out of 885-888]	[974]	[9751.1/0]	[out of 893-889]	[out of 839-842]	[out of 814-838]	[959-972]	[out of 809-881]	[out of 872-879]	[out of 880]			N	%
Bones [C40-41]	2	0	0	0	0	0	0	0	0	77	79	565	486	86.02
Digestive organs [C15-26]	4	19	0	0	3	479	140	1	2	59	707	1'684	977	58.02
Endocrine glands [C73-75]	3	0	0	0	0	165	1	0	0	0	169	414	245	59.18
Eye and adnexa [C69]	0	3	0	0	21	29	7	0	82	6	148	329	181	55.02
Female genital organs [C51-56]	13	3	1	0	1	21	2	0	0	18	59	423	364	86.05
Hematopoietic system [C42]	42	4	0	0	0	1	65	0	0	51	163	963	800	83.07
Ill defined [C76,80]	34	31	3	0	11	10	117	0	24	32	262	639	377	59
Intrathoracic organs (excl. lung) [C37-38]	3	2	0	0	0	3	26	0	0	6	40	240	200	83.33
Lip, oral cavity, pharynx [C00-14]	10	61	83	1	11	43	50	0	500	75	834	2'145	1'311	61.12
Lymph nodes [C77]	0	0	0	0	0	0	1'466	0	0	0	1'466	1'555	89	5.72
Male genital organs [C60-63]	23	70	6	0	2	137	4	1	4	9	256	1'836	1'580	86.06
Mammary gland [C50]	204	2	0	2'940	0	1'260	3	0	0	12	4'421	7'974	3'553	44.56
Nervous system [C47,70-72]	0	0	0	0	0	0	16	0	0	9	25	366	341	93.17
Nose, ear, sinuses, larynx [C30-32]	0	2	0	0	0	75	5	0	2	17	101	224	123	54.91
Peritoneum and retroperitoneum [C48]	5	1	0	0	0	0	3	0	0	0	9	33	24	72.73
Respiratory system [C33-34]	0	1	0	0	0	190	1	0	1	6	199	322	123	38.2
Skin [C44]	84	4'426	3'154	14	2'786	106	212	2'239	797	27	13'845	19'045	5'200	27.3
Soft tissue [C49]	6'689	143	0	0	0	2	8	0	0	1'043	7'885	11'092	3'207	28.91
Unknown [C80]	730	717	0	9	2	108	447	0	332	289	2'634	4'791	2'157	45.02
Urinary organs [C64-68]	1	0	0	0	0	40	12	0	0	7	60	346	286	82.66
Grand Total	7'847	5'485	3'247	2'964	2'837	2'669	2'585	2'241	1'744	1'743	33'362	54'986	21'624	60.67

N: number
