# Supplementary material for: Incidence rates of the most common canine tumors based on data from the Swiss Canine Cancer Registry (2008 to 2020)
Source: PLoS One. 2024 Apr 18;19(4):e0302231. doi: 10.1371/journal.pone.0302231 (PMC11025767; doi:10.1371/journal.pone.0302231)
Supplement: S5 Table — The tumor categories that formed the largest group per each topographical site are emphasized in bold. (PDF) [file pone.0302231.s005.pdf]

S5 Table. Absolute distribution of each morphologic tumor category per topographical site where *other tumors* accounted for more than 60% of all tumors, compared to the ten most common tumor types.

Tumor category [code]	Lip, oral cavity, pharynx [C00-14]	Male genital organs [C60-63]	Hematopoietic system [C42]	Bones [C40-41]	Female genital organs [C51-56]	Urinary organs [C64-68]	Nervous system [C47,70-72]	Intrathoracic organs (excl. lung) [C37-38]	Peritoneum and retroperitoneum [C48]	Unknown [C80]	Other topo-graphies	Grand Total
Neoplasms, NOS [800]	157	22	38	23	22	15	20	24	5	292	1'156	1'774
Epithelial neoplasms, NOS [801-804]	12	4	5	0	7	4	0	10	**6**	268	1'114	1'430
Squamous cell neoplasms [805-808]	334	10	0	0	9	8	0	0	0	320	2'023	2'704
Basal cell neoplasms [809-811]	0	1	0	0	0	0	0	0	0	0	2'305	2'306
Transitional cell papillomas and carcinomas [812-813]	0	6	0	0	0	**217**	0	0	0	23	24	270
Adenomas and adenocarcinomas [814-838]	43	137	1	0	42	44	0	4	0	142	3'733	4'146
Adnexal and skin appendage neoplasms [839-842]	23	3	0	0	1	0	0	0	0	6	4'388	4'421
Cystic, mucinous and serous neoplasms [844-849]	0	0	0	0	7	0	0	0	1	9	125	142
Ductal and lobular neoplasms [850-854]	0	0	0	0	3	0	0	0	0	3	194	200
Acinar cell neoplasms [855]	1	0	0	0	0	0	0	0	0	2	12	15
Thymic epithelial neoplasms [858]	0	0	0	0	0	0	0	42	0	0	0	42
Specialized gonadal neoplasms [859-867]	0	**995**	0	0	91	0	0	0	0	0	5	1'091
Paragangliomas and glomus tumors [868-871]	0	0	0	0	1	0	0	0	2	0	75	78
Melanocytoma and Melanomas [872-879]	**567**	12	0	0	1	0	0	0	0	475	1'842	2'897
Soft tissue tumors and sarcomas, NOS [880]	77	9	52	77	19	7	11	6	0	290	1'221	1'769
Fibromatous neoplasms [881-883]	44	9	7	6	34	1	0	1	1	193	1'749	2'045
Myxomatous neoplasms [884]	4	1	3	0	3	0	0	1	0	34	103	149
Lipomatous neoplasms [885-888]	13	23	44	2	13	1	0	3	5	752	7'098	7'954
Myomatous neoplasms [889-892]	1	2	4	0	**139**	22	0	0	1	43	123	335
Complex mixed and stromal neoplasms [893-899]	6	0	0	0	0	1	0	0	0	22	4'387	4'416
Fibroepithelial neoplasms [900-903]	0	0	0	0	0	0	0	0	0	5	15	20
Synovial-like neoplasms [904]	0	0	0	0	0	0	0	0	0	0	15	15
Mesothelial neoplasms [905]	0	0	0	0	0	0	0	16	2	4	6	28
Germ cell neoplasms [906-909]	0	491	0	0	17	0	0	0	1	0	1	510
Blood vessel tumors [912-916]	13	22	**615**	7	5	8	1	**101**	5	460	1'088	2'325
Lymphatic vessel tumors [917]	0	0	1	0	0	0	0	0	0	1	2	4
Osseous and chondromatous neoplasms [918-924]	46	0	2	**417**	0	1	0	0	0	20	80	566
Miscellaneous bone tumors [926]	0	0	0	1	0	0	0	0	0	0	0	1
Odontogenic tumors [927-934]	524	0	0	7	0	0	0	0	0	1	2	534
Miscellaneous tumors [935-937]	0	4	0	0	3	0	0	0	0	0	3	10
Gliomas [938-948]	0	0	0	0	0	0	**144**	0	0	1	0	145
Neuroepitheliomatous neoplasms [949-952]	0	0	0	0	0	0	4	0	0	1	4	9
Meningiomas [953]	0	0	0	0	0	0	87	0	0	0	0	87
Nerve sheath tumors [954-957]	0	0	0	0	0	0	66	1	0	0	0	67
Granular cell tumors [958]	10	0	0	0	0	0	0	0	0	0	0	10
Malignant lymphomas, NOS or diffuse [959-972]	28	4	63	0	2	12	16	25	3	446	1'772	2'371
Mature B-cell lymphomas [967-972]	0	0	2	0	0	0	0	1	0	1	2	6
Mature T- and NK-cell lymphomas [970-971]	22	0	0	0	0	0	0	0	0	0	186	208
Plasma cell neoplasms [973]	74	4	26	0	0	0	0	0	0	109	407	620
Mast cell neoplasms [974]	61	70	4	0	3	0	0	2	1	717	4'627	5'485
Neoplasms of histiocytes and accessory lymphoid cells [97	85	7	56	25	1	5	17	3	0	151	3'390	3'740
Immunoproliferative diseases [976]	0	0	0	0	0	0	0	0	0	0	1	1
Lymphoid leukemias [981-983]	0	0	27	0	0	0	0	0	0	0	0	27
Myeloid leukemias [984-993]	0	0	4	0	0	0	0	0	0	0	0	4
Myeloproliferative neoplasms [995-996]	0	0	9	0	0	0	0	0	0	0	0	9
Grand Total	2'145	1'836	963	565	423	346	366	240	33	4'791	43'278	54'986

The tumor categories that formed the largest group per each topographical site are emphasized in **bold.**
