# Supplementary material for: Incidence rates of the most common canine tumors based on data from the Swiss Canine Cancer Registry (2008 to 2020)
Source: PLoS One. 2024 Apr 18;19(4):e0302231. doi: 10.1371/journal.pone.0302231 (PMC11025767; doi:10.1371/journal.pone.0302231)
Supplement: S6 Table — IR: incidence rate (tumors per 100‘000 dog-years at risk); N: number; 95%CI: 95% confidence interval; [/0]: benign, [/1]: uncertain whether benign or malignant, [/2]: in situ, [/3]: malignant; B: biopsy, C: cytology, Ne: necropsy; NOS: not otherwise specified; *Preferred term indicated in the Vet-ICD-O-canine-1 was changed to a term more closely matching the tumor entity described in the diagnoses; **Code and/or term not available in Vet-ICD-O-canine-1 and assigned based on pathological tumor diagnosis. (PDF) [file pone.0302231.s006.pdf]

S6 Table. Morphological tumor diagnoses, absolute frequency, and incidence rates per 100'000 dog-years at risk of the 54'986 tumors registered in the Swiss Canine Cancer Registry, 2008–2020.

Tumor categories and tumors [code]	N tumors	IR (95%CI)	Number of cases with indicated						
			Behavior code				Diagnostic method		
			[/0]	[/1]	[/2]	[/3]	B	C	Ne
**Neoplasms, NOS [800]**	**1'774**	**24.88 (23.7-26.0)**	**44**	**940**	**0**	**790**	**787**	**931**	**56**
8000/0 Neoplasm, benign	42	0.59 (0.4-0.8)	42	0	0	0	26	16	0
8000/1 Neoplasm, uncertain whether benign or malignant	378	5.30 (4.8-5.9)	0	378	0	0	150	217	11
8000/3 Neoplasm, malignant	565	7.92 (7.3-8.6)	0	0	0	565	229	314	22
8004/1 Tumor, spindle cell type, NOS**	133	1.87 (1.6-2.2)	0	133	0	0	58	74	1
8004/3 Malignant tumor, spindle cell type	73	1.02 (0.8-1.3)	0	0	0	73	63	10	0
8006.1/0 Round cell tumor, benign**	2	0.03 (0.0-0.1)	2	0	0	0	0	2	0
8006.1/1 Round cell tumor, NOS	429	6.02 (5.5-6.6)	0	429	0	0	194	219	16
8006.1/3 Round cell tumor, malignant**	152	2.13 (1.8-2.5)	0	0	0	152	67	79	6
**Epithelial neoplasms, NOS [801-804]**	**1'430**	**20.06 (19.0-21.1)**	**0**	**77**	**162**	**1'191**	**957**	**428**	**45**
8010/1 Epithelial tumor, NOS**	77	1.08 (0.9-1.3)	0	77	0	0	28	48	1
8010/2 Carcinoma in situ, NOS	162	2.27 (1.9-2.6)	0	0	162	0	161	0	1
8010/3 Carcinoma, NOS	1'073	15.05 (14.2-16.0)	0	0	0	1'073	669	380	24
8012/3 Large cell carcinoma, NOS	2	0.03 (0.0-0.1)	0	0	0	2	0	0	2
8020/3 Carcinoma, undifferentiated, NOS	13	0.18 (0.1-0.3)	0	0	0	13	10	0	3
8021/34 Carcinoma, anaplastic, NOS	99	1.39 (1.1-1.7)	0	0	0	99	86	0	13
8032/3 Spindle cell carcinoma, NOS	3	0.04 (0.0-0.1)	0	0	0	3	3	0	0
8041/3 Small cell carcinoma, NOS	1	0.01 (0.0-0.1)	0	0	0	1	0	0	1
**Squamous cell neoplasms [805-808]**	**2'704**	**37.94 (36.5-39.4)**	**1'668**	**0**	**8**	**1'028**	**2'633**	**60**	**11**
8050/0 Papilloma, NOS	795	11.15 (10.4-11.9)	795	0	0	0	793	0	2
8050/3 Papillary carcinoma*	62	0.87 (0.7-1.1)	0	0	0	62	60	0	2
8052/0 Squamous cell papilloma	6	0.08 (0.0-0.2)	6	0	0	0	6	0	0
8053/0 Squamous cell papilloma, inverted	1	0.01 (0.0-0.1)	1	0	0	0	1	0	0
8070/2 Squamous cell carcinoma in situ	8	0.11 (0.0-0.2)	0	0	8	0	8	0	0
8070/3 Squamous cell carcinoma, NOS	966	13.55 (12.7-14.4)	0	0	0	966	900	59	7
8071.1/0 Nailbed keratoacanthoma	35	0.49 (0.3-0.7)	35	0	0	0	35	0	0
8071/0 Infundibular keratinizing acanthoma	831	11.65 (10.9-12.5)	831	0	0	0	830	1	0
**Basal cell neoplasms [809-811]**	**2'306**	**32.35 (31.0-33.7)**	**2'241**	**0**	**0**	**65**	**2'183**	**114**	**9**
8090/0 Basal cell tumor	207	2.90 (2.5-3.3)	207	0	0	0	118	89	0
8090/3 Basal cell carcinoma, NOS	34	0.48 (0.3-0.7)	0	0	0	34	32	1	1
8094/3 Basosquamous carcinoma	3	0.04 (0.0-0.1)	0	0	0	3	3	0	0
8100/0 Trichoepitelioma, NOS	746	10.46 (9.7-11.2)	746	0	0	0	740	0	6
8100/3 Trichoepithelioma, malignant	26	0.36 (0.2-0.5)	0	0	0	26	26	0	0
8101/0 Trichofolliculoma	25	0.35 (0.2-0.5)	25	0	0	0	24	0	1
8102.2/0 Tricholemmoma, inferior	1	0.01 (0.0-0.1)	1	0	0	0	1	0	0
8102/0 Tricholemmoma, NOS	15	0.21 (0.1-0.3)	15	0	0	0	15	0	0
8105.1/0 Trichoblastoma, NOS	691	9.69 (9.0-10.4)	691	0	0	0	667	24	0
8110/0 Pilomatricoma, NOS	556	7.80 (7.2-8.5)	556	0	0	0	555	0	1
8110/3 Pilomatricoma, malignant	2	0.03 (0.0-0.1)	0	0	0	2	2	0	0
**Transitional cell papillomas and carcinomas [812-813]**	**270**	**3.79 (3.3-4.3)**	**0**	**0**	**5**	**265**	**173**	**78**	**19**
8120/2 Urothelial carcinoma, in situ	5	0.07 (0.0-0.2)	0	0	5	0	5	0	0
8120/3 Transitional cell carcinoma, NOS*	264	3.70 (3.3-4.2)	0	0	0	264	167	78	19
8130/3 Papillary urothelial carcinoma	1	0.01 (0.0-0.1)	0	0	0	1	1	0	0
**Adenomas and adenocarcinomas [814-838]**	**4'146**	**58.15 (56.4-59.9)**	**1'467**	**10**	**4**	**2'665**	**3'541**	**294**	**311**
8140/0 Adenoma, NOS	1'134	15.90 (15.0-16.8)	1'134	0	0	0	1'028	17	89
8140/2 Adenocarcinoma in situ*	4	0.06 (0.0-0.1)	0	0	4	0	4	0	0
8140/3 Adenocarcinoma, NOS	2'234	31.33 (30.0-32.6)	0	0	0	2'234	1'893	216	125
8150/3 Islet cell carcinoma, NOS	35	0.49 (0.3-0.7)	0	0	0	35	33	0	2
8151/0 Insulinoma, NOS	13	0.18 (0.1-0.3)	13	0	0	0	5	0	8
8151/3 Insulinoma, malignant	11	0.15 (0.1-0.3)	0	0	0	11	9	0	2
8160/0 Cholangiocellular adenoma	2	0.03 (0.0-0.1)	2	0	0	0	1	0	1
8160/3 Cholangiocarcinoma	13	0.18 (0.1-0.3)	0	0	0	13	8	0	5
8161/0 Bile duct cystadenoma	2	0.03 (0.0-0.1)	2	0	0	0	0	0	2
8161/3 Bile duct cystadenocarcinoma	1	0.01 (0.0-0.1)	0	0	0	1	1	0	0
8170/0 Hepatoma, benign	48	0.67 (0.5-0.9)	48	0	0	0	29	0	19
8170/1 Hepatocellular neoplasm, NOS**	1	0.01 (0.0-0.1)	0	1	0	0	1	0	0
8170/3 Hepatocellular carcinoma, NOS	143	2.01 (1.7-2.4)	0	0	0	143	84	50	9
8174/3 Hepatocellular carcinoma, clear cell type	1	0.01 (0.0-0.1)	0	0	0	1	1	0	0
8190/3 Trabecular carcinoma	2	0.03 (0.0-0.1)	0	0	0	2	2	0	0
8211/0 Tubular adenoma	94	1.32 (1.1-1.6)	94	0	0	0	94	0	0
8211/3 Tubular carcinoma	45	0.63 (0.5-0.8)	0	0	0	45	44	0	1
8230/3 Solid carcinoma	14	0.20 (0.1-0.3)	0	0	0	14	14	0	0
8246/1 Neuroendocrine neoplasm, NOS**	8	0.11 (0.0-0.2)	0	8	0	0	5	1	2
8246/3 Neuroendocrine carcinoma, NOS	28	0.39 (0.3-0.6)	0	0	0	28	15	3	10
8260/0 Papillary adenoma	133	1.87 (1.6-2.2)	133	0	0	0	131	0	2
8260/3 Papillary adenocarcinoma	9	0.13 (0.1-0.2)	0	0	0	9	8	0	1
8261/0 Villous adenoma, NOS	1	0.01 (0.0-0.1)	1	0	0	0	1	0	0
8263/0 Tubulopapillary adenoma	35	0.49 (0.3-0.7)	35	0	0	0	33	0	2
8263/3 Tubulopapillary adenocarcinoma	45	0.63 (0.5-0.8)	0	0	0	45	42	0	3
8265/3 Micropapillary adenocarcinoma	3	0.04 (0.0-0.1)	0	0	0	3	3	0	0
8270/0 Chromophobe adenoma	1	0.01 (0.0-0.1)	1	0	0	0	0	0	1
8272/3 Pituitary carcinoma, NOS	3	0.04 (0.0-0.1)	0	0	0	3	1	0	2
8290/0 Oxyphilic adenoma	2	0.03 (0.0-0.1)	2	0	0	0	1	0	1
8312/3 Renal cell carcinoma, NOS	23	0.32 (0.2-0.5)	0	0	0	23	16	6	1
8314/3 Lipid-rich carcinoma	1	0.01 (0.0-0.1)	0	0	0	1	1	0	0
8330/0 Follicular adenoma, NOS	1	0.01 (0.0-0.1)	1	0	0	0	1	0	0
8330/3 Follicular carcinoma, NOS	7	0.10 (0.0-0.2)	0	0	0	7	3	0	4
8345/1 C cell neoplasm, NOS**	1	0.01 (0.0-0.1)	0	1	0	0	1	0	0
8345/3 C cell carcinoma	4	0.06 (0.0-0.1)	0	0	0	4	4	0	0

8370/0 Adrenal cortical adenoma	1	0.01 (0.0-0.1)	1	0	0	0	0	0	1
8370/3 Adrenal cortical carcinoma	43	0.60 (0.4-0.8)	0	0	0	43	24	1	18
**Adnexal and skin appendage neoplasms [839-842]**	**4'421**	**62.00 (60.1-63.8)**	**2'837**	**955**	**0**	**629**	**3'750**	**656**	**15**
8401.2/0 Anal sac adenoma	3	0.04 (0.0-0.1)	3	0	0	0	2	0	1
8401.2/1 Anal sac neoplasm, NOS**	1	0.01 (0.0-0.1)	0	1	0	0	0	1	0
8401.2/3 Anal sac adenocarcinoma	434	6.09 (5.5-6.7)	0	0	0	434	203	227	4
8401/0 Apocrine adenoma	20	0.28 (0.2-0.4)	20	0	0	0	20	0	0
8401/1 Apocrine neoplasm, NOS**	2	0.03 (0.0-0.1)	0	2	0	0	2	0	0
8401/3 Apocrine adenocarcinoma	16	0.22 (0.1-0.4)	0	0	0	16	15	0	1
8402/3 Clear cell adnexal carcinoma	1	0.01 (0.0-0.1)	0	0	0	1	1	0	0
8410.1/0 Meibomian adenoma	320	4.49 (4.0-5.0)	320	0	0	0	319	0	1
8410.1/1 Meibomian gland epithelioma	102	1.43 (1.2-1.7)	0	102	0	0	102	0	0
8410.1/3 Meibomian gland adenocarcinoma	1	0.01 (0.0-0.1)	0	0	0	1	1	0	0
8410.2/0 Hepatoid adenoma	1'442	20.22 (19.2-21.3)	1'442	0	0	0	1'283	158	1
8410.2/1 Hepatoid gland epithelioma	48	0.67 (0.5-0.9)	0	48	0	0	45	3	0
8410.2/1 Hepatoid gland neoplams, NOS*	234	3.28 (2.9-3.7)	0	234	0	0	20	214	0
8410.2/3 Hepatoid gland adenocarcinoma	125	1.75 (1.5-2.1)	0	0	0	125	88	35	2
8410/0 Sebaceous adenoma	1'046	14.67 (13.8-15.6)	1'046	0	0	0	1'039	4	3
8410/1 Sebaceous epithelioma	567	7.95 (7.3-8.6)	0	567	0	0	552	14	1
8410/3 Sebaceous adenocarcinoma	41	0.57 (0.4-0.8)	0	0	0	41	40	0	1
8420/0 Ceruminous adenoma	6	0.08 (0.0-0.2)	6	0	0	0	6	0	0
8420/1 Ceruminous neoplasm, NOS**	1	0.01 (0.0-0.1)	0	1	0	0	1	0	0
8420/3 Ceruminous adenocarcinoma	11	0.15 (0.1-0.3)	0	0	0	11	11	0	0
**Cystic, mucinous and serous neoplasms [844-849]**	**142**	**1.99 (1.7-2.3)**	**78**	**0**	**1**	**63**	**133**	**1**	**8**
8440/0 Cystic adenoma	78	1.09 (0.9-1.4)	78	0	0	0	77	0	1
8440/3 Cystic adenocarcinoma	4	0.06 (0.0-0.1)	0	0	0	4	4	0	0
8480/3 Mucinous carcinoma	17	0.24 (0.1-0.4)	0	0	0	17	14	0	3
8490/2 Signet-ring cell carcinoma in situ**	1	0.01 (0.0-0.1)	0	0	1	0	1	0	0
8490/3 Signet-ring cell carcinoma	42	0.59 (0.4-0.8)	0	0	0	42	37	1	4
**Ductal and lobular neoplasms [850-854]**	**200**	**2.80 (2.4-3.2)**	**95**	**0**	**5**	**100**	**199**	**0**	**1**
8500/0 Ductal adenoma	54	0.76 (0.6-1.0)	54	0	0	0	54	0	0
8500/2 Ductal carcinoma in situ**	4	0.06 (0.0-0.1)	0	0	4	0	4	0	0
8500/3 Ductal carcinoma, NOS	44	0.62 (0.4-0.8)	0	0	0	44	44	0	0
8501/3 Comedocarcinoma	25	0.35 (0.2-0.5)	0	0	0	25	24	0	1
8503/0 Intraductal papillary adenoma	41	0.57 (0.4-0.8)	41	0	0	0	41	0	0
8503/2 Intraductal papillary carcinoma in situ**	1	0.01 (0.0-0.1)	0	0	1	0	1	0	0
8503/3 Intraductal papillary carcinoma	30	0.42 (0.3-0.6)	0	0	0	30	30	0	0
8530/3 Inflammatory mammary carcinoma	1	0.01 (0.0-0.1)	0	0	0	1	1	0	0
**Ductal and lobular neoplasms [850-854]**	**15**	**0.21 (0.1-0.3)**	**0**	**0**	**0**	**15**	**15**	**0**	**0**
8550/3 Acinar adenocarcinoma	1	0.01 (0.0-0.1)	0	0	0	1	1	0	0
8560/3 Adenosquamous carcinoma	14	0.20 (0.1-0.3)	0	0	0	14	14	0	0
**Thymic epithelial neoplasms [858]**	**42**	**0.59 (0.4-0.8)**	**0**	**0**	**0**	**42**	**27**	**8**	**7**
8580/3 Thymoma, NOS	42	0.59 (0.4-0.8)	0	0	0	42	27	8	7
**Specialized gonadal neoplasms [859-867]**	**1'091**	**15.30 (14.4-16.2)**	**3**	**995**	**0**	**93**	**1'031**	**10**	**50**
8590/1 Sex cord-gonadal stromal tumor, NOS	2	0.03 (0.0-0.1)	0	2	0	0	1	0	1
8610/0 Luteoma, NOS	3	0.04 (0.0-0.1)	3	0	0	0	2	0	1
8620/3 Granulosa cell tumor, NOS	91	1.28 (1.0-1.6)	0	0	0	91	89	0	2
8640/1 Sertoli cell tumor, NOS	315	4.42 (3.9-4.9)	0	315	0	0	299	2	14
8640/3 Sertoli cell tumor, malignant**	2	0.03 (0.0-0.1)	0	0	0	2	2	0	0
8650/1 Interstitial cell tumor, NOS	678	9.51 (8.8-10.2)	0	678	0	0	638	8	32
**Paragangliomas and glomus tumors [868-871]**	**78**	**1.09 (0.9-1.4)**	**2**	**15**	**0**	**61**	**31**	**1**	**46**
8680/3 Paraganglioma	2	0.03 (0.0-0.1)	0	0	0	2	0	0	2
8691/1 Aortic body tumor, NOS	16	0.22 (0.1-0.4)	0	15	0	1	4	1	11
8692/3 Carcinoma of the carotid body	2	0.03 (0.0-0.1)	0	0	0	2	1	0	1
8700/3 Pheochromocytoma, NOS	56	0.79 (0.6-1.0)	0	0	0	56	24	0	32
8711/0 Glomus tumor	2	0.03 (0.0-0.1)	2	0	0	0	2	0	0
**Melanocytoma and Melanomas [872-879]**	**2'897**	**40.63 (39.1-42.1)**	**1'071**	**82**	**0**	**1'744**	**2'689**	**194**	**14**
8720.0/0 Melanocytoma	1'068	14.98 (14.1-15.9)	1'068	0	0	0	1'037	25	6
8720/1 Melanocytic neoplasm, NOS**	82	1.15 (0.9-1.4)	0	82	0	0	60	22	0
8720/3 Melanoma, NOS	1'622	22.75 (21.6-23.9)	0	0	0	1'622	1'472	143	7
8722/3 Balloon cell melanoma	1	0.01 (0.0-0.1)	0	0	0	1	1	0	0
8726.1/0 Melanoacanthoma	3	0.04 (0.0-0.1)	3	0	0	0	3	0	0
8730/3 Amelanotic melanoma	121	1.70 (1.4-2.0)	0	0	0	121	116	4	1
**Soft tissue tumors and sarcomas, NOS [880]**	**1'769**	**24.81 (23.7-26.0)**	**1**	**25**	**0**	**1'743**	**1'014**	**724**	**31**
8800.0/1 Soft tissue tumor, NOS	25	0.35 (0.2-0.5)	0	25	0	0	10	14	1
8800/0 Soft tissue tumor, benign	1	0.01 (0.0-0.1)	1	0	0	0	0	0	1
8800/3 Sarcoma, NOS	1'054	14.78 (13.9-15.7)	0	0	0	1'054	603	432	19
8801/3 Spindle cell sarcoma, NOS	684	9.59 (8.9-10.3)	0	0	0	684	397	278	9
8805/3 Undifferentiated sarcoma	5	0.07 (0.0-0.2)	0	0	0	5	4	0	1
**Fibromatous neoplasms [881-883]**	**2'045**	**28.68 (27.4-29.9)**	**353**	**1'270**	**0**	**422**	**2'033**	**5**	**7**
8810/0 Fibroma, NOS	344	4.82 (4.3-5.4)	344	0	0	0	342	0	2
8810/3 Fibrosarcoma, NOS	411	5.76 (5.2-6.3)	0	0	0	411	406	1	4
8811/0 Fibromyxoma	3	0.04 (0.0-0.1)	3	0	0	0	3	0	0
8815.1/1 Canine perivascular wall tumor, NOS	1'270	17.81 (16.8-18.8)	0	1'270	0	0	1'265	4	1
8815.1/3 Canine perivascular wall tumor, malignant**	8	0.11 (0.0-0.2)	0	0	0	8	8	0	0
8830/3 Malignant fibrous histiocytoma	3	0.04 (0.0-0.1)	0	0	0	3	3	0	0
8832/0 Dermatofibroma	6	0.08 (0.0-0.2)	6	0	0	0	6	0	0
**Fibromatous neoplasms [881-883]**	**149**	**2.09 (1.8-2.5)**	**15**	**0**	**0**	**134**	**144**	**2**	**3**
8840/0 Myxoma, NOS	15	0.21 (0.1-0.3)	15	0	0	0	14	0	1
8840/3 Myxosarcoma, NOS	134	1.88 (1.6-2.2)	0	0	0	134	130	2	2
**Lipomatous neoplasms [885-888]**	**7'954**	**111.56 (109.0-114.0)**	**7'847**	**0**	**0**	**107**	**2'106**	**5'765**	**83**
8850/0 Lipoma, NOS	7'627	106.97 (104.5-109.3)	7'627	0	0	0	1'798	5'761	68
8850/3 Liposarcoma, NOS	107	1.50 (1.2-1.8)	0	0	0	107	102	4	1
8851/0 Fibrolipoma	141	1.98 (1.7-2.3)	141	0	0	0	141	0	0
8856/0 Infiltrative lipoma	17	0.24 (0.1-0.4)	17	0	0	0	16	0	1

8857/0 Spindle cell lipoma	2	0.03 (0.0-0.1)	2	0	0	0	2	0	0
8861/0 Angiolipoma	7	0.10 (0.0-0.2)	7	0	0	0	7	0	0
8862/0 Chondrolipoma	12	0.17 (0.1-0.3)	12	0	0	0	11	0	1
8870/0 Myelolipoma	41	0.57 (0.4-0.8)	41	0	0	0	29	0	12
**Myomatous neoplasms [889-892]**	**335**	**4.70 (4.2-5.2)**	**238**	**0**	**0**	**97**	**305**	**1**	**29**
8890.1/0 Fibroleiomyoma	4	0.06 (0.0-0.1)	4	0	0	0	4	0	0
8890.4/0 Lipoleiomyoma	1	0.01 (0.0-0.1)	1	0	0	0	1	0	0
8890/0 Leiomyoma, NOS	232	3.25 (2.8-3.7)	232	0	0	0	204	1	27
8890/3 Leiomyosarcoma, NOS	87	1.22 (1.0-1.5)	0	0	0	87	86	0	1
8900/0 Rhabdomyoma, NOS	1	0.01 (0.0-0.1)	1	0	0	0	1	0	0
8900/3 Rhabdomyosarcoma, NOS	9	0.13 (0.1-0.2)	0	0	0	9	9	0	0
8910/3 Embryonal rhabdomyosarcoma	1	0.01 (0.0-0.1)	0	0	0	1	0	0	1
**Complex mixed and stromal neoplasms [893-899]**	**4'416**	**61.94 (60.1-63.7)**	**2'964**	**325**	**3**	**1'124**	**4'367**	**24**	**25**
8936/3 Gastrointestinal stromal tumor (GIST)	21	0.29 (0.2-0.4)	0	0	0	21	18	0	3
8940/0 Benign mixed tumor, NOS	2'373	33.28 (31.9-34.6)	2'373	0	0	0	2'361	2	10
8940/1 Mixed tumor, NOS**	319	4.47 (4.0-5.0)	0	319	0	0	305	10	4
8940/3 Mixed carcinoma, NOS	686	9.62 (8.9-10.4)	0	0	0	686	685	0	1
8941.1/3 Carcinoma arising in a complex adenoma/benign mixed tumor	12	0.17 (0.1-0.3)	0	0	0	12	12	0	0
8960/3 Nephroblastoma, NOS	1	0.01 (0.0-0.1)	0	0	0	1	1	0	0
8980/3 Carcinosarcoma, NOS	28	0.39 (0.3-0.6)	0	0	0	28	27	1	0
8982/0 Myoepithelioma	18	0.25 (0.1-0.4)	18	0	0	0	18	0	0
8982/3 Malignant myoepithelioma	14	0.20 (0.1-0.3)	0	0	0	14	14	0	0
8983.1/2 Complex carcinoma in situ**	3	0.04 (0.0-0.1)	0	0	3	0	3	0	0
8983.1/3 Complex carcinoma	362	5.08 (4.6-5.6)	0	0	0	362	355	3	4
8983/0 Complex adenoma	573	8.04 (7.4-8.7)	573	0	0	0	565	5	3
8983/1 Complex neoplasm, NOS**	6	0.08 (0.0-0.2)	0	6	0	0	3	3	0
**Fibroepithelial neoplasms [900-903]**	**20**	**0.28 (0.2-0.4)**	**20**	**0**	**0**	**0**	**20**	**0**	**0**
9010/0 Fibroadenoma, NOS	20	0.28 (0.2-0.4)	20	0	0	0	20	0	0
**Synovial-like neoplasms [904]**	**15**	**0.21 (0.1-0.3)**	**0**	**0**	**0**	**15**	**15**	**0**	**0**
9040/3 Synovial cell sarcoma	15	0.21 (0.1-0.3)	0	0	0	15	15	0	0
**Mesothelial neoplasms [905]**	**28**	**0.39 (0.3-0.6)**	**0**	**0**	**0**	**28**	**22**	**2**	**4**
9050/3 Mesothelioma, malignant, NOS	28	0.39 (0.3-0.6)	0	0	0	28	22	2	4
**Germ cell neoplasms [906-909]**	**510**	**7.15 (6.5-7.8)**	**0**	**501**	**0**	**9**	**492**	**1**	**17**
9060/3 Dysgerminoma	8	0.11 (0.0-0.2)	0	0	0	8	8	0	0
9061/1 Seminoma, NOS	490	6.87 (6.3-7.5)	0	490	0	0	474	1	15
9061/3 Seminoma, malignant**	1	0.01 (0.0-0.1)	0	0	0	1	1	0	0
9073/1 Gonadoblastoma	1	0.01 (0.0-0.1)	0	1	0	0	1	0	0
9080/1 Teratoma, NOS	10	0.14 (0.1-0.3)	0	10	0	0	8	0	2
**Blood vessel tumors [912-916]**	**2'325**	**32.61 (31.3-33.9)**	**1'035**	**0**	**0**	**1'290**	**2'095**	**1**	**229**
9120/0 Hemangioma, NOS	1'010	14.16 (13.3-15.1)	1'010	0	0	0	1'003	0	7
9120/3 Hemangiosarcoma, NOS	1'290	18.09 (17.1-19.1)	0	0	0	1'290	1'067	1	222
9121/0 Cavernous hemangioma	1	0.01 (0.0-0.1)	1	0	0	0	1	0	0
9141/0 Angiokeratoma	22	0.31 (0.2-0.5)	22	0	0	0	22	0	0
9160/0 Angiofibroma	2	0.03 (0.0-0.1)	2	0	0	0	2	0	0
**Lymphatic vessel tumors [917]**	**4**	**0.06 (0.0-0.1)**	**1**	**0**	**0**	**3**	**4**	**0**	**0**
9170/0 Lymphangioma	1	0.01 (0.0-0.1)	1	0	0	0	1	0	0
9170/3 Lymphangiosarcoma	3	0.04 (0.0-0.1)	0	0	0	3	3	0	0
**Osseous and chondromatous neoplasms [918-924]**	**566**	**7.94 (7.3-8.6)**	**26**	**0**	**0**	**540**	**502**	**28**	**36**
9180.1/3 Osteoblastic osteosarcoma	3	0.04 (0.0-0.1)	0	0	0	3	0	0	3
9180/0 Osteoma, NOS	9	0.13 (0.1-0.2)	9	0	0	0	6	0	3
9180/3 Osteosarcoma, NOS	404	5.67 (5.1-6.2)	0	0	0	404	356	28	20
9181/3 Chondroblastic osteosarcoma	3	0.04 (0.0-0.1)	0	0	0	3	1	0	2
9193/3 Periosteal osteosarcoma	1	0.01 (0.0-0.1)	0	0	0	1	0	0	1
9210.1/3 Multilobular tumor of bone	14	0.20 (0.1-0.3)	0	0	0	14	13	0	1
9220/0 Chondroma, NOS	17	0.24 (0.1-0.4)	17	0	0	0	14	0	3
9220/3 Chondrosarcoma, NOS	115	1.61 (1.3-1.9)	0	0	0	115	112	0	3
**Miscellaneous bone tumors [926]**	**1**	**0.01 (0.0-0.1)**	**1**	**0**	**0**	**0**	**1**	**0**	**0**
9262/0 Ossifying fibroma	1	0.01 (0.0-0.1)	1	0	0	0	1	0	0
**Odontogenic tumors [927-934]**	**534**	**7.49 (6.9-8.1)**	**518**	**15**	**0**	**1**	**534**	**0**	**0**
9270.1/3 Ameloblastic carcinoma	1	0.01 (0.0-0.1)	0	0	0	1	1	0	0
9270/1 Odontogenic tumor, NOS**	14	0.20 (0.1-0.3)	0	14	0	0	14	0	0
9280/0 Odontoma, NOS	1	0.01 (0.0-0.1)	1	0	0	0	1	0	0
9290/0 Ameloblastic fibro-odontoma	1	0.01 (0.0-0.1)	1	0	0	0	1	0	0
9310.2/1 Acanthomatous ameloblastoma	1	0.01 (0.0-0.1)	0	1	0	0	1	0	0
9310/0 Ameloblastoma, NOS	208	2.92 (2.5-3.3)	208	0	0	0	208	0	0
9322/0 Peripheral odontogenic fibroma**	308	4.32 (3.8-4.8)	308	0	0	0	308	0	0
**Miscellaneous tumors [935-937]**	**10**	**0.14 (0.1-0.3)**	**10**	**0**	**0**	**0**	**3**	**4**	**3**
9374.1/0 Canine transmissible venereal tumor	10	0.14 (0.1-0.3)	10	0	0	0	3	4	3
**Gliomas [938-948]**	**145**	**2.03 (1.7-2.4)**	**6**	**4**	**0**	**135**	**99**	**0**	**46**
9380/3 Glioma, NOS	5	0.07 (0.0-0.2)	0	0	0	5	2	0	3
9382/3 Oligoastrocytoma, NOS	10	0.14 (0.1-0.3)	0	0	0	10	8	0	2
9390/0 Choroid plexus papilloma	6	0.08 (0.0-0.2)	6	0	0	0	3	0	3
9390/1 Choroid plexus tumor	1	0.01 (0.0-0.1)	0	1	0	0	0	0	1
9390/3 Choroid plexus carcinoma	12	0.17 (0.1-0.3)	0	0	0	12	10	0	2
9391/1 Cellular ependymoma	2	0.03 (0.0-0.1)	0	2	0	0	0	0	2
9400.0/3 Gliomatosis cerebri	17	0.24 (0.1-0.4)	0	0	0	17	15	0	2
9400/3 Astrocytoma, NOS	20	0.28 (0.2-0.4)	0	0	0	20	13	0	7
9421/1 Pilocytic astrocytoma	1	0.01 (0.0-0.1)	0	1	0	0	0	0	1
9440/3 Glioblastoma	14	0.20 (0.1-0.3)	0	0	0	14	4	0	10
9450/3 Oligodendroglioma, NOS	56	0.79 (0.6-1.0)	0	0	0	56	44	0	12
9473/3 CNS embryonal tumor, NOS	1	0.01 (0.0-0.1)	0	0	0	1	0	0	1
**Neuroepitheliomatous neoplasms [949-952]**	**9**	**0.13 (0.1-0.2)**	**0**	**2**	**0**	**7**	**8**	**0**	**1**
9490/1 Ganglioneuroma	1	0.01 (0.0-0.1)	0	1	0	0	1	0	0
9500/3 Neuroblastoma, NOS	5	0.07 (0.0-0.2)	0	0	0	5	4	0	1
9501/3 Medulloepithelioma	2	0.03 (0.0-0.1)	0	0	0	2	2	0	0

9505/1 Ganglioglioma, NOS	1	0.01 (0.0-0.1)	0	1	0	0	1	0	0
**Meningiomas [953]**	**87**	**1.22 (1.0-1.5)**	**0**	**79**	**0**	**8**	**47**	**0**	**40**
9530.0/1 Meningioma, NOS	23	0.32 (0.2-0.5)	0	23	0	0	16	0	7
9530.1/1 Meningioma, NOS, WHO grade I	8	0.11 (0.0-0.2)	0	8	0	0	8	0	0
9530.1/3 Rhabdoid meningioma	1	0.01 (0.0-0.1)	0	0	0	1	1	0	0
9530.2/1 Microcystic meningioma	1	0.01 (0.0-0.1)	0	1	0	0	0	0	1
9530/3 Meningioma, malignant; WHO grade III	5	0.07 (0.0-0.2)	0	0	0	5	5	0	0
9531/1 Meningothelial meningioma	4	0.06 (0.0-0.1)	0	4	0	0	1	0	3
9532/1 Fibrous meningioma	7	0.10 (0.0-0.2)	0	7	0	0	3	0	4
9533/1 Psammomatous meningioma	3	0.04 (0.0-0.1)	0	3	0	0	1	0	2
9537/1 Transitional meningioma	25	0.35 (0.2-0.5)	0	25	0	0	6	0	19
9537/3 Transitional meningioma, malignant**	1	0.01 (0.0-0.1)	0	0	0	1	0	0	1
9539/1 Meningioma, WHO grade II	8	0.11 (0.0-0.2)	0	8	0	0	6	0	2
9539/3 Meningeal sarcomatosis	1	0.01 (0.0-0.1)	0	0	0	1	0	0	1
**Nerve sheath tumors [954-957]**	**67**	**0.94 (0.7-1.2)**	**26**	**27**	**0**	**14**	**61**	**0**	**6**
9540.0/0 Schwannoma, NOS	25	0.35 (0.2-0.5)	25	0	0	0	23	0	2
9540.0/3 Schwannoma, malignant**	2	0.03 (0.0-0.1)	0	0	0	2	2	0	0
9540.2/3 Malignant peripheral nerve sheath tumor, NOS	12	0.17 (0.1-0.3)	0	0	0	12	9	0	3
9540/0 Neurofibroma, NOS	1	0.01 (0.0-0.1)	1	0	0	0	1	0	0
9563/1 Nerve sheath tumor, NOS	27	0.38 (0.2-0.6)	0	27	0	0	26	0	1
**Granular cell tumors [958]**	**10**	**0.14 (0.1-0.3)**	**9**	**0**	**0**	**1**	**10**	**0**	**0**
9580/0 Granular cell tumor, NOS	9	0.13 (0.1-0.2)	9	0	0	0	9	0	0
9580/3 Granular cell tumor, malignant	1	0.01 (0.0-0.1)	0	0	0	1	1	0	0
**Malignant lymphomas, NOS or diffuse [959-972]**	**2'371**	**33.26 (31.9-34.6)**	**0**	**0**	**0**	**2'371**	**581**	**1'725**	**65**
9591.1/3 B-cell lymphoma, NOS	260	3.65 (3.2-4.1)	0	0	0	260	142	114	4
9591.2/3 T-cell lymphoma, NOS**	188	2.64 (2.3-3.0)	0	0	0	188	116	59	13
9591/3 Malignant lymphoma (non-Hodgkin), NOS	1'923	26.97 (25.8-28.2)	0	0	0	1'923	323	1'552	48
**Mature B-cell lymphomas [967-972]**	**6**	**0.08 (0.0-0.2)**	**0**	**0**	**0**	**6**	**4**	**0**	**2**
9673/3 Mantle cell lymphoma	1	0.01 (0.0-0.1)	0	0	0	1	1	0	0
9688/3 T-cell-rich large B-cell lymphoma	1	0.01 (0.0-0.1)	0	0	0	1	0	0	1
9690/3 Follicular lymphoma, NOS	2	0.03 (0.0-0.1)	0	0	0	2	2	0	0
9699/3 Marginal zone lymphoma, NOS	2	0.03 (0.0-0.1)	0	0	0	2	1	0	1
**Mature T- and NK-cell lymphomas [970-971]**	**208**	**2.92 (2.5-3.3)**	**0**	**0**	**0**	**208**	**204**	**3**	**1**
9700/3 Cutaneous epitheliotropic lymphoma	192	2.69 (2.3-3.1)	0	0	0	192	191	0	1
9702.1/3 T-zone lymphoma (TZL), nodal	14	0.20 (0.1-0.3)	0	0	0	14	12	2	0
9702/3 Peripheral T-cell lymphoma, NOS	2	0.03 (0.0-0.1)	0	0	0	2	1	1	0
**Plasma cell neoplasms [973]**	**620**	**8.69 (8.0-9.4)**	**396**	**200**	**0**	**24**	**514**	**92**	**14**
9732/3 Plasma cell myeloma	23	0.32 (0.2-0.5)	0	0	0	23	7	7	9
9734/0 Plasmacytoma, extramedullary (cutaneous)	396	5.55 (5.0-6.1)	396	0	0	0	339	57	0
9734/1 Plasmacytoma, extramedullary (digestive tract)	83	1.16 (0.9-1.4)	0	83	0	0	76	6	1
9734/1 Plasmacytoma, NOS**	117	1.64 (1.4-2.0)	0	117	0	0	92	21	4
9734/3 Plasmacytoma, extramedullary (malignant)	1	0.01 (0.0-0.1)	0	0	0	1	0	1	0
**Mast cell neoplasms [974]**	**5'485**	**76.96 (74.9-78.9)**	**0**	**5'310**	**0**	**175**	**4'081**	**1'393**	**11**
9740.0/1 Subcutaneous mast cell tumor	142	1.99 (1.7-2.3)	0	142	0	0	142	0	0
9740.0/3 Subcutaneous mast cell tumor, metastatic**	5	0.07 (0.0-0.2)	0	0	0	5	5	0	0
9740.1/1 Cutaneous mast cell tumor grade Patnaik I	221	3.10 (2.7-3.5)	0	221	0	0	221	0	0
9740.2/1 Cutaneous mast cell tumor grade Patnaik II	1'935	27.15 (25.9-28.4)	0	1'935	0	0	1'894	40	1
9740.3/1 Cutaneous mast cell tumor grade Patnaik III	97	1.36 (1.1-1.7)	0	97	0	0	82	15	0
9740.4/1 Cutaneous mast cell tumor grade Kiupel low	130	1.82 (1.5-2.2)	0	130	0	0	86	44	0
9740.5/1 Cutaneous mast cell tumor grade Kiupel high	31	0.43 (0.3-0.6)	0	31	0	0	26	5	0
9740.6/1 Cutaneous mast cell tumor grade Patnaik I, Kiupel low	107	1.50 (1.2-1.8)	0	107	0	0	107	0	0
9740.7/1 Cutaneous mast cell tumor grade Patnaik II, Kiupel low	1'021	14.32 (13.4-15.2)	0	1'021	0	0	1'015	6	0
9740.8/1 Cutaneous mast cell tumor grade Patnaik II, Kiupel high	68	0.95 (0.7-1.2)	0	68	0	0	68	0	0
9740.9/1 Cutaneous mast cell tumor grade Patnaik III, Kiupel high	74	1.04 (0.8-1.3)	0	74	0	0	73	1	0
9740/1 Mast cell tumor, NOS	1'484	20.81 (19.8-21.9)	0	1'484	0	0	290	1'187	7
9740/3 Mast cell tumor, metastatic**	168	2.36 (2.0-2.7)	0	0	0	168	72	93	3
9741.1/3 Systemic mastocytosis, NOS	2	0.03 (0.0-0.1)	0	0	0	2	0	2	0
**Neoplasms of histiocytes and accessory lymphoid cells [975]**	**3'740**	**52.52 (50.7-54.1)**	**3'247**	**0**	**0**	**493**	**2'596**	**1'058**	**86**
9751.1/0 Canine cutaneous histiocytoma	3'247	45.59 (44.0-47.1)	3'247	0	0	0	2'407	840	0
9755/3 Histiocytic sarcoma	493	6.91 (6.3-7.5)	0	0	0	493	189	218	86
**Immunoproliferative diseases [976]**	**1**	**0.01 (0.0-0.1)**	**0**	**1**	**0**	**0**	**0**	**0**	**1**
9766/1 Lymphomatoid granulomatosis	1	0.01 (0.0-0.1)	0	1	0	0	0	0	1
**Lymphoid leukemias [981-983]**	**27**	**0.38 (0.2-0.6)**	**0**	**0**	**0**	**27**	**2**	**19**	**6**
9823/3 B-cell chronic lymphocytic leukemia/small (cell) lymphocytic lymphoma	1	0.01 (0.0-0.1)	0	0	0	1	0	1	0
9835/3 Precursor cell lymphoblastic leukemia, NOS	26	0.36 (0.2-0.5)	0	0	0	26	2	18	6
**Myeloid leukemias [984-993]**	**4**	**0.06 (0.0-0.1)**	**0**	**0**	**0**	**4**	**1**	**1**	**2**
9860/3 Myeloid leukemia, NOS	4	0.06 (0.0-0.1)	0	0	0	4	1	1	2
**Myeloproliferative neoplasms [995-996]**	**9**	**0.13 (0.1-0.2)**	**0**	**0**	**0**	**9**	**9**	**0**	**0**
9961/3 Primary myelofibrosis	9	0.13 (0.1-0.2)	0	0	0	9	9	0	0
Grand Total	54'986	775.34 (764.2-777.1)	26'219	10'833	188	17'746	40'023	13'623	1'340

IR: incidence rate (tumors per 100‘000 dog-years at risk); N: number; 95%CI: 95% confidence interval; [/0]: benign, [/1]: uncertain whether benign or malignant, [/2]: in situ, [/3]: malignant; B: biopsy, C: cytology, Ne: necropsy; NOS: not otherwise specified; *Preferred term indicated in the Vet-ICD-O-canine-1 was changed to a term more closely matching the tumor entity described in the diagnoses; **Code and/or term not available in Vet-ICD-O-canine-1 and assigned based on pathological tumor diagnosis.
