# Supplementary material for: Incidence rates of the most common canine tumors based on data from the Swiss Canine Cancer Registry (2008 to 2020)
Source: PLoS One. 2024 Apr 18;19(4):e0302231. doi: 10.1371/journal.pone.0302231 (PMC11025767; doi:10.1371/journal.pone.0302231)
Supplement: S7 Table — IRR: incidence rate ratio; 95%CI: 95% confidence interval; Std. Error: standard error; N: number; (na.omit): number of tumors/DYAR after excluding cases/dogs with missing information on one or more variables; DYAR: dog-years at risk; AIC: Akaike information criterion. (PDF) [file pone.0302231.s007.pdf]

S7 Table. Results from the negative binomial regression model showing the incidence rate ratios of 21’803 malignant tumors in the Swiss Canine Cancer Registry (2008-2020), abosulte tumor numbers, and dog-years at risk for each value and measures of goodness of fit.

Variable	Value	Estimate	IRR	Std. Error	Z-value	P-value	N malignant tumors	N malignant tumors (na.omit)	DYAR Amicus (na.omit)
	(Intercept)	-8.16	0.00	0.10	-83.11	<0.05	-	-	-
Age group	0-3	-	-	-	-	-	671	607	1'719'573
	4-7	1.81	6.10	0.07	24.53	<0.05	4'987	4'629	1'930'543
	8-11	2.90	18.24	0.07	40.25	<0.05	12'255	11'350	1'642'359
	12-15	2.75	15.58	0.07	36.99	<0.05	5'533	5'062	913'152
	16-19	1.40	4.04	0.13	10.55	<0.05	180	155	136'959
Sex	Female	-	-	-	-	-	13'928	12'709	3'268'330
	Male	-0.30	0.74	0.04	-7.68	<0.05	9'830	9'094	3'074'256
Breed group and section	Mixed breed	-	-	-	-	-	5'527	5'415	2'135'579
	Sheepdogs (Group 1, Section 1)	0.12	1.13	0.12	1.05	0.296	2'228	2'162	748'513
	Cattledogs (except Swiss Cattledogs) (Group 1, Section 2)	1.34	3.81	0.23	5.94	<0.05	37	37	2'852
	Pinscher and Schnauzer type (Group 2, Section 1)	1.02	2.78	0.13	8.13	<0.05	722	699	103'058
	Molossian type (Group 2, Section 2)	1.20	3.33	0.12	10.02	<0.05	1'799	1'752	267'345
	Swiss Mountain- and Cattledogs (Group 2, Section 3)	0.76	2.14	0.12	6.24	<0.05	997	978	212'920
	Large and medium sized Terriers (Group 3, Section 1)	0.45	1.57	0.13	3.41	<0.05	431	422	102'049
	Small sized Terriers (Group 3, Section 2)	0.12	1.13	0.12	1.03	0.304	1'039	1'021	343'072
	Bull type Terriers (Group 3, Section 3)	0.88	2.42	0.14	6.41	<0.05	284	272	51'849
	Toy Terriers (Group 3, Section 4)	-0.38	0.68	0.13	-2.92	<0.05	405	392	229'844
	Dachshunds (Group 4)	0.19	1.21	0.14	1.39	0.163	265	257	74'187
	Nordic sledge dogs (Group 5, Section 1)	0.26	1.30	0.14	1.87	0.062	269	261	77'738
	Nordic hunting dogs (Group 5, Section 2)	2.09	8.08	0.39	5.41	<0.05	9	9	276
	Nordic Watchdogs and Herders (Group 5, Section 3)	0.97	2.64	0.36	2.67	<0.05	10	10	966
	European Spitz (Group 5, Section 4)	-0.23	0.79	0.18	-1.27	0.202	67	67	34'259
	Asian Spitz and related breeds (Group 5, Section 5)	0.75	2.12	0.16	4.63	<0.05	123	121	21'560
	Primitive type (Group 5, Section 6)	1.30	3.68	0.30	4.30	<0.05	17	16	1'295
	Primitive type - hunting dogs (Group 5, Section 7)	0.93	2.53	0.20	4.55	<0.05	50	50	7'448
	Scent Hounds (Group 6, Section 1)	0.32	1.38	0.13	2.47	<0.05	390	383	117'504
	Leash (scent) Hounds (Group 6, Section 2)	0.63	1.87	0.22	2.84	<0.05	36	36	7'171
	Related breeds (Group 6, Section 3)	1.26	3.54	0.13	9.50	<0.05	380	374	49'277
	Continental pointing dogs (Group 7, Section 1)	1.01	2.74	0.13	7.55	<0.05	380	368	55'773
	British and Irish Pointers and Setters (Group 7, Section 2)	1.01	2.74	0.14	7.32	<0.05	327	323	41'953
	Retrievers (Group 8, Section 1)	0.90	2.45	0.11	7.85	<0.05	3'574	3'484	593'229
	Flushing dogs (Group 8, Section 2)	0.59	1.80	0.13	4.63	<0.05	635	616	126'908
	Water dogs (Group 8, Section 3)	0.64	1.90	0.15	4.18	<0.05	135	133	34'340
	Bichons and related breeds (Group 9, Section 1)	-0.31	0.73	0.13	-2.32	<0.05	305	296	178'135
	Poodle (Group 9, Section 2)	-0.09	0.91	0.13	-0.68	0.494	350	345	140'249
	Small Belgian dogs (Group 9, Section 3)	2.31	10.04	1.10	2.09	<0.05	1	1	30
	Hairless dogs (Group 9, Section 4)	0.52	1.68	0.31	1.65	0.098	16	14	2'760
	Tibetan breeds (Group 9, Section 5)	-0.09	0.91	0.14	-0.66	0.512	268	263	108'792
	Chihuahueno (Group 9, Section 6)	-0.87	0.42	0.16	-5.59	<0.05	148	146	162'451
	English toy Spaniels (Group 9, Section 7)	-0.23	0.79	0.17	-1.37	0.170	83	83	41'894
	Japan Chin and Pekingese (Group 9, Section 8)	-0.31	0.73	0.22	-1.41	0.158	38	38	15'231
	Continental toy Spaniel and others (Group 9, Section 9)	0.06	1.07	0.18	0.35	0.728	70	70	20'098
	Kromfohrländer (Group 9, Section 10)	0.91	2.48	0.35	2.59	<0.05	11	11	1'121
	Small molossian type dogs (Group 9, Section 11)	0.87	2.38	0.13	6.86	<0.05	651	636	186'506
	Long-haired or fringed Sighthounds (Group 10, Section 1)	1.24	3.45	0.19	6.59	<0.05	73	72	7'154
	Rough-haired Sighthounds (Group 10, Section 2)	1.93	6.88	0.26	7.49	<0.05	27	25	2'084
	Short-haired Sighthounds (Group 10, Section 3)	0.49	1.63	0.15	3.18	<0.05	147	145	35'116

Signif. codes: 0 ‘***’ 0.001 ‘**’ 0.01 ‘*’ 0.05 ‘.’ 0.1 ‘ ’ 1
(Dispersion parameter for Negative Binomial(4.737) family taken to be 1)

Null deviance: 3651.95 on 963 degrees of freedom
Residual deviance: 928.25 on 919 degrees of freedom
AIC: 5791.5

Number of Fisher Scoring iterations: 1

Theta: 4.737
Std. Err.: 0.330

2 x log-likelihood: -5699.463

IRR: incidence rate ratio; 95%CI: 95% confidence interval; Std. Error: standard error; N: number; (na.omit): number of tumors/DYAR after excluding cases/dogs with missing information on one or more variables; DYAR: dog-years at risk; AIC: Akaike information criterion.
