# Supplementary material for: Incidence rates of the most common canine tumors based on data from the Swiss Canine Cancer Registry (2008 to 2020)
Source: PLoS One. 2024 Apr 18;19(4):e0302231. doi: 10.1371/journal.pone.0302231 (PMC11025767; doi:10.1371/journal.pone.0302231)
Supplement: S8 Table — IR: incidence rate (tumors per 100‘000 dog-years at risk); N: number; 95%CI: 95% confidence interval; DYAR: dog-years at risk. (PDF) [file pone.0302231.s008.pdf]

S8 Table. The 20 Swiss dog breeds (precise breed) with the highest incidence rates for benign tumors between 2008 and 2020 and their respective Swiss Canine Cancer Registry data.

Dog breed (precise)	N dogs Amicus	DYAR Amicus	N benign tumors	DYAR benign tumors	IR benign tumors (95%CI)
Polski Owczarek Nizinny	369	2'749	62	352	2'349 (1'729-2'891)
Field Spaniel	201	1'598	31	223	1'969 (1'318-2'754)
Magyar Vizsla	2'419	17'373	324	2'043	1'896 (1'667-2'079)
Russian Black Terrier	324	2'038	35	226	1'712 (1'196-2'388)
Flat Coated Retriever	6'169	40'615	673	3'673	1'689 (1'534-1'787)
Airedale Terrier	1'556	10'772	170	1'021	1'601 (1'350-1'834)
Doberman Pinscher	2'452	14'110	215	1'032	1'546 (1'327-1'742)
Irish Soft Coated Wheaten Terrier	668	5'308	76	511	1'457 (1'128-1'792)
Schnauzer - Standard	1'405	10'244	146	926	1'445 (1'203-1'676)
Gordon Setter	1'570	10'900	155	976	1'435 (1'207-1'664)
Rhodesian Ridgeback	3'772	24'922	352	2'270	1'416 (1'269-1'568)
Irish Terrier	765	5'512	76	489	1'394 (1'086-1'726)
Nova Scotia Duck Tolling Retriever	1'636	10'744	147	1'057	1'357 (1'156-1'608)
Schnauzer - Giant	2'423	16'097	216	1'320	1'352 (1'169-1'533)
Boxer	8'336	53'684	625	3'205	1'182 (1'075-1'259)
Dogo Argentino	712	4'265	48	263	1'132 (830-1'492)
Briard	1'363	8'971	94	546	1'056 (847-1'282)
Bobtail (Old English Sheepdog)	1'218	8'423	86	550	1'026 (817-1'261)
King Charles Spaniel	406	2'516	25	123	1'006 (643-1'467)
Bouvier des Flandres	367	2'364	23	112	988 (617-1'460)
Grand Total	1'032'029	7'135'182	29'179	178'534	410 (404-414)

IR: incidence rate (tumors per 100‘000 dog-years at risk); N: number; 95%CI: 95% confidence interval; DYAR: dog-years at risk.
