# Supplementary material for: Incidence rates of the most common canine tumors based on data from the Swiss Canine Cancer Registry (2008 to 2020)
Source: PLoS One. 2024 Apr 18;19(4):e0302231. doi: 10.1371/journal.pone.0302231 (PMC11025767; doi:10.1371/journal.pone.0302231)
Supplement: S9 Table — IR: incidence rate (tumors per 100‘000 dog-years at risk); N: number; 95%CI: 95% confidence interval; DYAR: dog-years at risk. (PDF) [file pone.0302231.s009.pdf]

S9 Table. The 20 Swiss dog breeds (precise breed) with the highest incidence rates for malignant tumors between 2008 and 2020 and their respective Swiss Canine Cancer Registry data.

Dog breed (precise)	N dogs Amicus	DYAR Amicus	N malignant tumors	DYAR malignant tumors	IR malignant tumors (95%CI)
Schnauzer - Giant	2'423	16'097	235	1'499	1'466 (1'279-1'659)
Boxer	8'336	53'684	765	4'280	1'442 (1'326-1'530)
Schnauzer - Standard	1'405	10'244	130	824	1'285 (1'060-1'507)
Rhodesian Ridgeback	3'772	24'922	318	2'071	1'278 (1'140-1'424)
Nova Scotia Duck Tolling Retriever	1'636	10'744	134	899	1'245 (1'045-1'477)
Doberman Pinscher	2'452	14'110	165	857	1'177 (998-1'362)
Bouvier des Flandres	367	2'364	27	115	1'171 (753-1'662)
Flat Coated Retriever	6'169	40'615	469	2'767	1'164 (1'053-1'264)
Magyar Vizsla	2'419	17'373	196	1'279	1'137 (976-1'298)
Dogo Argentino	712	4'265	46	285	1'076 (790-1'439)
Gordon Setter	1'570	10'900	115	701	1'065 (871-1'266)
Airedale Terrier	1'556	10'772	105	636	983 (797-1'180)
Irish Terrier	765	5'512	53	364	965 (720-1'258)
Manchester Terrier	226	1'657	16	129	959 (552-1'568)
Dogue de Bordeaux	796	4'736	42	251	887 (639-1'199)
King Charles Spaniel	406	2'516	22	135	875 (548-1'324)
Polski Owczarek Nizinny	369	2'749	23	140	846 (530-1'255)
Rottweiler	5'333	30'033	244	1'335	813 (714-921)
Field Spaniel	201	1'598	13	104	813 (433-1'391)
Scottish Terrier	980	6'843	55	340	809 (605-1'046)
Grand Total	1'032'029	7'135'182	24'076	151'019	338 (333-342)

IR: incidence rate (tumors per 100‘000 dog-years at risk); N: number; 95%CI: 95% confidence interval; DYAR: dog-years at risk.
